# StainAI: quantitative mapping of stained microglia and insights into brain-wide neuroinflammation and therapeutic effects in cardiac arrest

**DOI:** 10.1038/s42003-025-07926-y

**Published:** 2025-03-20

**Authors:** Chao-Hsiung Hsu, Yi-Yu Hsu, Be-Ming Chang, Katherine Raffensperger, Micah Kadden, Hoai T. Ton, Essiet-Adidiong Ette, Stephen Lin, Janiya Brooks, Mark W. Burke, Yih-Jing Lee, Paul C. Wang, Michael Shoykhet, Tsang-Wei Tu

**Affiliations:** 1 Molecular Imaging Laboratory, Department of Radiology, Howard University, Washington, DC USA; 2 Miin Wu School of Computing, National Cheng Kung University, Tainan City, Taiwan; 3 Center for Neuroscience Research, Children’s National Research Institute, Washington, DC USA; 4 Pediatric Critical Care Medicine, Children’s National Hospital, Washington, DC USA; 5 Department of Physiology and Biophysics, Howard University, Washington, DC USA; 6 School of Medicine, Fu-Jen Catholic University, New Taipei City, Taiwan; 7 Department of Physics, Fu-Jen Catholic University, New Taipei City, Taiwan; 8 Department of Pediatrics, George Washington University School of Medicine and Health Sciences, Washington, DC USA

**Keywords:** Image processing, Inflammasome, Glial development

## Abstract

Microglia, the brain’s resident macrophages, participate in development and influence neuroinflammation, which is characteristic of multiple brain pathologies. Diverse insults cause microglia to alter their morphology from “resting” to “activated” shapes, which vary with stimulus type, brain location, and microenvironment. This morphologic diversity commonly restricts microglial analyses to specific regions and manual methods. We introduce StainAI, a deep learning tool that leverages 20x whole-slide immunohistochemistry images for rapid, high-throughput analysis of microglial morphology. StainAI maps microglia to a brain atlas, classifies their morphology, quantifies morphometric features, and computes an activation score for any region of interest. As a proof of principle, StainAI was applied to a rat model of pediatric asphyxial cardiac arrest, accurately classifying millions of microglia across multiple slices, surpassing current methods by orders of magnitude, and identifying both known and novel activation patterns. Extending its application to a non-human primate model of simian immunodeficiency virus infection further demonstrated its generalizability beyond rodent datasets, providing new insights into microglial responses across species. StainAI offers a scalable, high-throughput solution for microglial analysis from routine immunohistochemistry images, accelerating research in microglial biology and neuroinflammation.

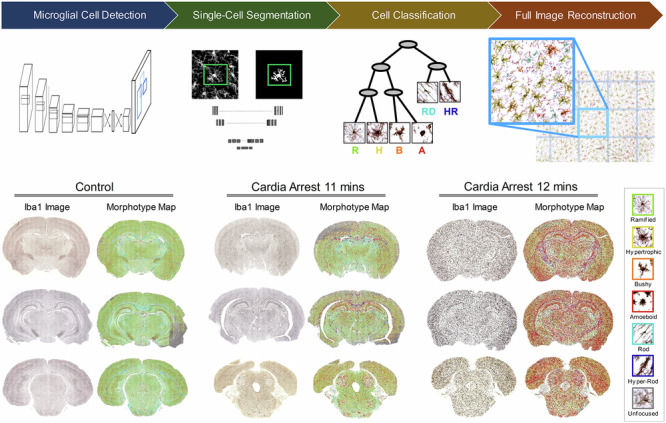

## Abstract (web summary)

StainAI enables high-throughput microglial analysis, mapping and quantifying morphology across entire brain regions, demonstrating scalability and cross-species adaptability in rodent and primate models.

## Introduction

Microglia, the brain's resident immune cells, play key roles in brain development, immune response, injury, and repair^1^. Under normal and pathologic states, microglia exhibit variable morphology^2^, which has been the subject of several, well-established classification schema^3–5^. Morphological analyses and classification of microglia from histologic sections stained with immunohistochemistry (IHC) or immunofluorescence constitute a major tool in studying microglial behavior and response to injury^6–8^. The variability in morphology and limited access to advanced microscopy tools, however, present multiple analytic challenges. Consequently, many studies continue to rely on time-consuming manual cell segmentation and classification^8–10^, which circumscribes regions of interest and limits sample size.

Several semi- and fully automated approaches to morphological analysis and classification of microglia have been proposed^11–16^. Semi-automated approaches still require significant human input. Fully automated rule-based methods have difficulty with histologic artifacts such as uneven staining intensity, debris, out-of-focus regions, and varying slice thickness^13–18^. More recently, deep learning-based methods using convolutional neural networks (CNNs)^19^ or UNet^20^ have improved proficiency in quantifying microglia^11,12,21–23^. These methods excel at distinguishing nuclear and somatic components from background but struggle adapting to heterogeneous cells and their features within diverse microenvironments across brain regions^24^. As a result, CNN- and UNet-based pipelines perform best with 3D stacks of confocal images^14–17^ and/or high-magnification (40x or greater) images with limited field of view (FOV)^25–27^. Neither imaging approach permits automated detection, localization, morphological analysis and classification of microglia across the whole brain at cellular resolution.

In the present study, we develop StainAI to overcome these limitations. Created using a multi-stage deep learning approach, StainAI integrates detection, segmentation, and classification models to quantify microglial morphology from low-power (20x) microscopic images of 40 µm-thick, whole brain slices stained with IHC for microglial marker of ionized calcium-binding adapter molecule 1 (Iba1). It then uses sequential slices mapped to a brain atlas to build a 3D map of microglial distribution across an entire rat brain. A key resource enabling StainAI is a comprehensive, high-quality ground truth dataset of 88,897 single-cell masks encompassing multiple brain regions and microglial activation states.

As proof-of-principle, StainAI was utilized to analyze microglial activation in a rat model of pediatric asphyxial cardiac arrest and therapeutic hypothermia^28^. In this model, as in children, a global insult—complete cessation of blood flow associated with cardiac arrest—paradoxically leads to focal hotspots of brain injury and microglial activation^29^. The hippocampal cornu ammonis 1 (CA1) region and the thalamic reticular nucleus (nRT) are known examples of such hotspots^28–30^. The model thus incorporates both negative (sham-arrested rats) and known positive (CA1 and nRT in arrested rats) controls, providing a fruitful testing ground for StainAI. Results show that StainAI accurately and efficiently identifies known and reveals novel microglial activation patterns across multiple brain regions in immature rats after asphyxial cardiac arrest and resuscitation. Finally, the StainAI pipeline was applied to an archival image of a pediatric rhesus macaque brain infected with Simian Immunodeficiency Virus macaque strain 251 (SIVmac251)^31,32^, demonstrating its generalizability in analyzing microglia across species.

## Results

### StainAI framework

**Fig. 1:**
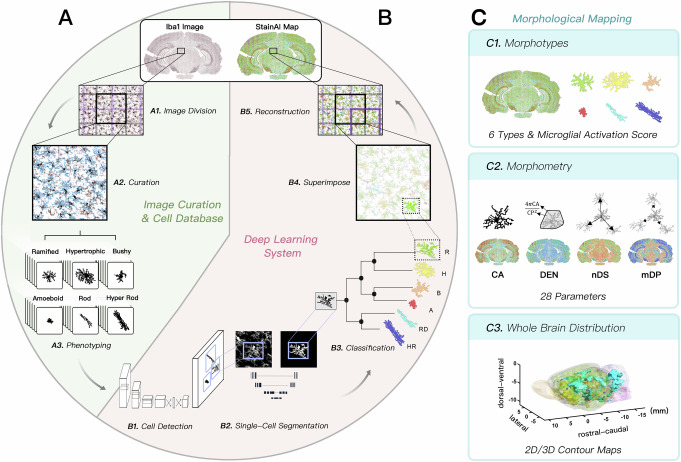
Framework of the StainAI system for microglial image analysis. **A** Pipeline for Iba1 image curation and microglial cell database development. A sub-image with a reduced field of view (FOV) of 238 × 238 μm^2^ was extracted for curation (**A1**). Raters manually outlined each cell boundary (**A2**) and classified each cell’s activation state into one of six morphotypes: ramified (R), hypertrophic (H), bushy (B), ameboid (A), rod-shaped (RD), and hypertrophic rod-shaped (HR) (**A3**). **B** Multi-stage deep learning system for processing whole-brain morphological maps. Each sub-image was first processed using YOLO for cell detection (**B1**), followed by UNet segmentation within bounding boxes expanded to 119 × 119 μm^2^ to capture the cell body and processes of a single cell (**B2**). A C5.0 decision tree classifier categorized the cells’ activation states based on morphometric parameters derived from the UNet segmentation (**B3**). The resulting single-cell masks were superimposed on the image (**B4**), with each cell color-coded according to its morphological phenotype (R: green, H: yellow, B: orange, A: red, RD: cyan, HR: blue). Panel B5 shows the reconstruction of a full-sized Iba1 image from two diagonally shifted sub-image units (black and purple boxes), reducing computational demands and minimizing errors in large image analysis. **C** The system produces maps displaying microglial morphotypes (**C1**), morphometric parameters (**C2**), and whole brain contours (**C3**), illustrating their distribution across the entire brain.

The StainAI pipeline was implemented in three distinct phases (Fig. 1). Phase 1 comprised image pre-processing and curation followed by development of a ground truth dataset containing reconstructions of 88,897 microglia by trained human observers (Fig. 1A, Supplementary Fig. 1). Phase 2 employed a multi-stage deep learning approach (Fig. 1B). First, a YOLO^33^-based object detection deep learning model identified microglial cells and generated bounding boxes to define their territorial domains. A UNet^24^ model then segmented the detected cells and created corresponding cell masks (Supplementary Fig. 2). For each mask, Brenner’s^34^ focus measure was computed, along with mean shortest distances between soma and between processes of adjacent cells, and a set of 25 morphometric parameters (Supplementary Fig. 3). Then, C5.0 decision tree classifier then assigned the morphometric parameter set for each cell to a morphological phenotype: ramified (R), hypertrophic (H), bushy (B), ameboid (A), rod-shaped (RD), and hypertrophic rod-shaped (HR). In Phase 2’s final step, each cell mask was mapped back onto the image with class assignment signified by color (Fig. 1B). Phase 3 registered 2D images to a rat brain atlas and segmented them into anatomic regions (Fig. 1C). For each region, it then quantified activation class and morphometric parameter densities. In the last step, Phase 3 interpolated each class’ and parameter’s density across contiguous anatomic regions in adjacent 2D slices, creating a 3D, whole-brain contour map of activation class and morphometric parameter distributions.

### StainAI performance

**Fig. 2:**
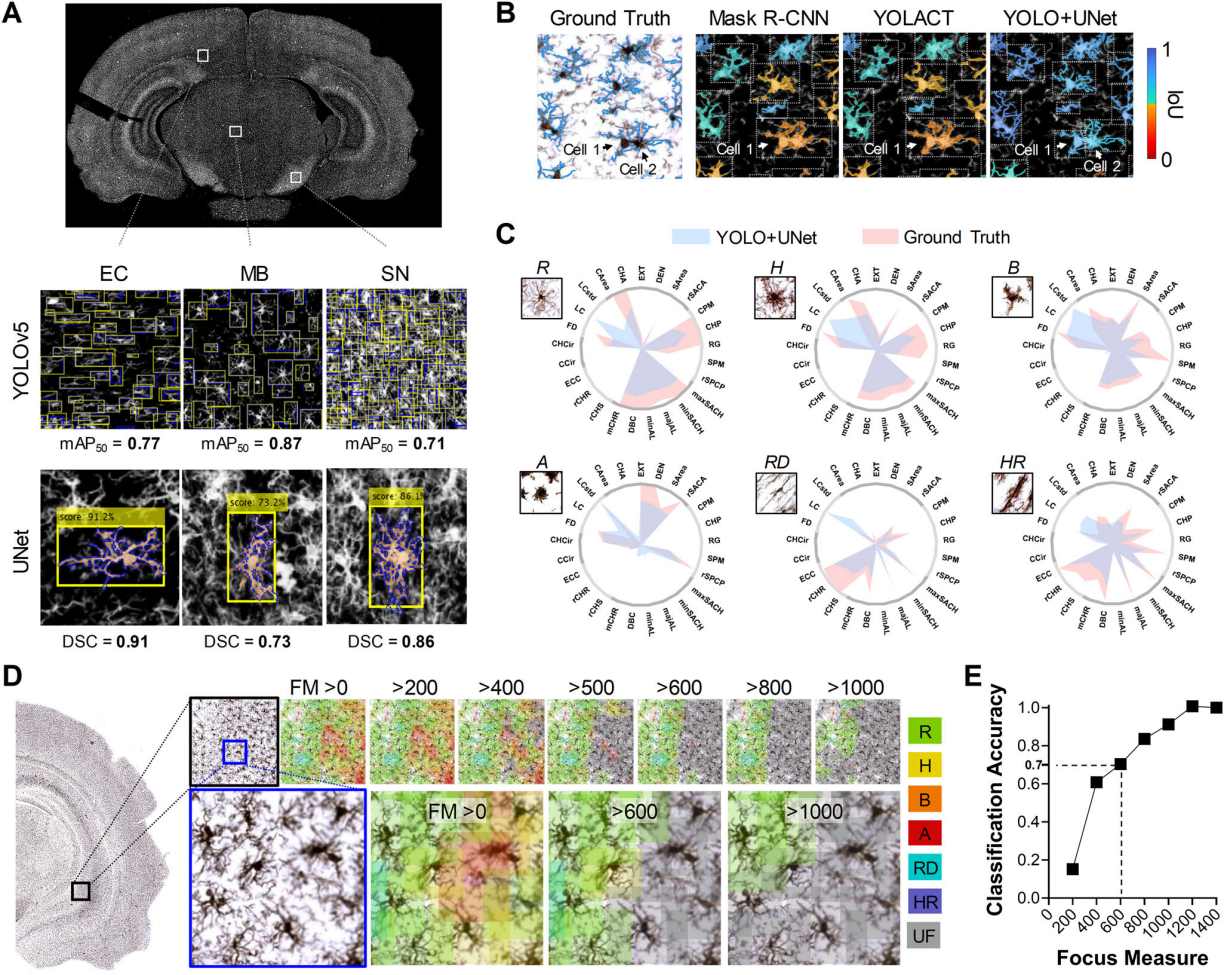
Model comparison and outcome evaluations. **A** A comparison of the system's performance in analyzing microglia across regions with varying cell densities and morphotypes, such as the external capsule (EC), midbrain (MB), and substantia nigra (SN), shows strong agreement with the ground truth (blue outlines) in both cell detection (yellow boxes) and segmentation (pink fills). **B** The StainAI system captured intricate details of microglial morphology and accurately differentiated overlapping cells (Cell 1 and Cell 2, indicated by arrows), with high agreement to the ground truth. In contrast to the smoother cell profiles detected by Mask R-CNN and YOLACT, StainAI provided more precise segmentation. Cell masks are color-coded based on their level of agreement with the ground truth, measured by Intersection over Union (IoU). **C** Radar charts compare the average single-cell segmentation between the ground truth and StainAI across six microglial activation states: (R), hypertrophic (H), bushy (B), ameboid (A), rod-shaped (RD), and hypertrophic rod-shaped (HR) using 25 morphometric parameters. The results show strong agreement, except for the fractal parameters lacunarity (LC) and standard deviation of lacunarity (stdLC), which capture the fine structural details of microglial processes. **D** Classification errors increased when the focus measure threshold was set lower, causing ramified cells (green) to be misclassified as inflamed types (yellow, orange, and red) due to blurry boundaries. **E** To reduce classification errors from low focus quality, a threshold of focus measure > 600, corresponding to 70% classification accuracy, was used to exclude cell images from analysis. mAP50: Mean Average Precision at 50% IoU; DSC: Dice Similarity Coefficient; FM: focus measure; UF: Unfocused cells identified by focus measure threshold. Refer to Supplementary Information for definitions of the morphometric parameters in (**C**).

StainAI’s YOLO+UNet pipeline detected and segmented microglia across multiple brain regions with high precision and accuracy (Fig. 2). For detection, it achieved a mean average precision at 50% Intersection over Union (IoU) threshold (mAP_50_) of 0.796, while for segmentation it achieved a mean Dice Similarity Coefficient (DSC) of 0.807 (Table 1). The pipeline performed well in regions with sparse and dense microglial populations (e.g., midbrain (MB) and substantia nigra (SN), respectively), and in the white matter with unique rod-shaped microglia (Fig. 2A and Table 1). YOLO+UNet outperformed two other deep learning pipelines, Mask R-CNN, and YOLACT, in detection and, to a greater extent, in segmentation (Fig. 2B and Table 2). The morphometric parameters calculated by YOLO+UNet closely resembled ground truth across all six morphological classes, albeit with a few deviations in fractal measures of finer processes (Fig. 2C).

**Table 1:** System performance for region-specific microglia detection and segmentation

Brain Region	Cell Detection	Cell Segmentation
	(mAP_50_)	(DSC)
Cortex	0.871	0.802
External Capsule	0.774	0.783
Midbrain	0.769	0.831
Diencephalon	0.689	0.819
CA1	0.839	0.817
CA2	0.855	0.803
CA3	0.794	0.799
Dentate Gyrus	0.875	0.788
Substantia Nigra	0.725	0.781
Internal Capsule	0.745	0.794
Hindbrain	0.741	0.839
Pituitary Gland	0.808	0.811
Whole Brain Average	0.796	0.807

Cellular data were collected from slice sections of both control (*n* = 1290) and injured (*n *= 1319) brains located at Bregma −6.2 mm.

*mAP*_50_ mean average precision with a 50% intersection over union, *DSC* Dice similarity coefficient.

**Table 2:** Comparative evaluation of microglial detection and segmentation methods

	Cell Detection		Cell Segmentation	
Model	Control	Injured	Control	Injured
Mask R-CNN	0.695	0.686	0.336	0.541
YOLACT	0.663	0.640	0.309	0.508
YOLO+UNet	0.762	0.715	0.763	0.712

The performance of Mask R-CNN, YOLACT, and YOLO+UNet was evaluated by their mean average precision at a 50% intersection over union (mAP_50_) using a dataset comprising 138 microglial images annotated from both control (*n* = 72) and injured (*n* = 66) brains.

The application of Brenner’s focus measure reduced classification errors caused by out-of-focus cells. The accuracy of the C5.0 decision tree classifier improved as the focus measure increased (Fig. 2D, E). When the focus measure exceeded 600, the model achieved high classification accuracy (>0.7) while excluding a small proportion of microglia (~4.3%). After removing poorly focused cells, the C5.0 classifier attained a Cohen’s kappa of 0.608 and a weighted average accuracy, precision, recall, and F1-score of 0.709, 0.729, 0.729, and 0.713, respectively, across all morphological classes (Table 3). The C5.0 decision tree classifier also outperformed multiple other deep learning-based classification algorithms on accuracy and F1 (Supplementary Table 1).

**Table 3:** System performance in classifying microglia throughout various brain regions

Morphotype	Precision	Recall	F1 Score	Accuracy
Ramified	0.745	0.894	0.812	0.671
Hypertrophic	0.654	0.356	0.461	0.624
Bushy	0.656	0.713	0.683	0.774
Ameboid	0.874	0.874	0.874	0.897
Rod	0.801	0.838	0.836	0.881
Hyper-Rod	0.720	0.507	0.595	0.744
Weighted Average	0.729	0.729	0.713	0.709

Cellular data were collected from slice sections of both control (*n *= 1290) and injured (*n *= 1319) brains located at Bregma −6.2 mm.

### StainAI application to brain injury

The trained StainAI pipeline was applied to a well-established model of pediatric asphyxial cardiac arrest and resuscitation in developing rats^28,29^. We also included a group of severely injured rats (arrest duration = 12.5 min) that were treated with mild therapeutic hypothermia (34 °C) for 8 h after resuscitation. In this cardiac arrest model, microglial activation in the hippocampus^35^ and cerebral cortex^10^, as well as the beneficial effect of mild post-arrest hypothermia^29^, have been well characterized. However, the extent of microglial activation and the effect of hypothermia in other brain regions remain unknown. This model thus provided robust positive and negative controls to evaluate StainAI performance and presented an opportunity for discovery.

#### Analysis of whole-brain slices with StainAI

**Fig. 3:**
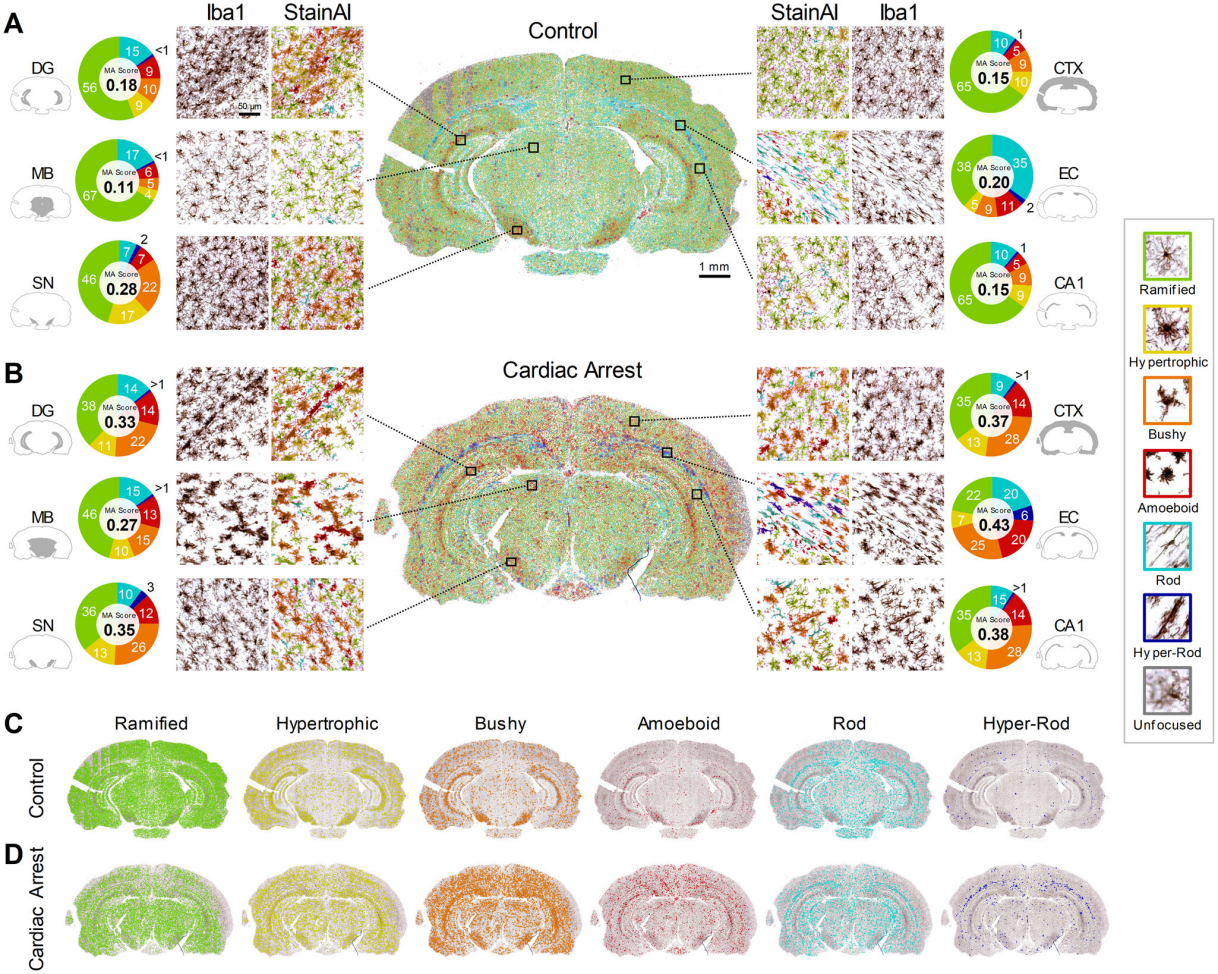
Example analysis of Iba1 images from control and cardiac arrest brains using StainAI. Morphotype maps generated by the StainAI pipeline for a control brain (**A**, **C**) and a cardiac arrest brain (**B**, **D**) are shown. Each microglial cell was segmented, color-coded into six distinct classes, and evaluated for focus quality as indicated in the legend. The pie charts show the percentage distribution of each cell class and the corresponding microglial activation score for each region in (**A** and **B**). The cardiac arrest brain (**B**), subjected to a 12-minute hypoxic-ischemic insult, exhibited a significantly increased density of activated microglia throughout the brain. The comprehensive mapping of six cell classes reveals region-specific changes in microglial morphotypes across the brain following injury (**C**, **D**). MA: microglial activation, DG: dentate gyrus, MB: midbrain, SN: substantia nigra, CTX: cortex, EC: external capsule, CA1: cornu ammonis 1.

StainAI analyzed 288 whole-brain slice images obtained with a 20x objective (image size ~ 12.6 × 7.5 mm, final magnification 250x) from sixteen rat brains across four cohorts: control (Control, *n* = 3), 11 min cardiac arrest (CA11, *n* = 3), 12 min cardiac arrest (CA12, *n* = 5), and 12.5 min cardiac arrest with hypothermia treatment (CA+HT, *n *= 5). Approximately eight million (8,038,454) cells were analyzed. Figure 3 shows examples of quantitative 2D maps generated by StainAI. In Control rats, ramified microglia predominated in all regions except in the external capsule (EC) where 35% are rod-shaped (Fig. 3A). StainAI also captured variability in microglial morphology across brain regions, with hypertrophic microglia, for instance, ranging from 4% in the MB to 17% in the SN.

The morphological map was visually and quantitatively different in a brain slice from a CA12 rat (Fig. 3B). The proportion of microglia with features of activation increased in all regions but to varying extents. For example, the percentage of bushy microglia tripled in the cerebral cortex but increased by ~20% in the SN. In the hippocampus, ameboid microglia tripled in frequency in CA1 but increased by only ~56% in the dentate gyrus. Figure 3C and D further highlight region-specific changes in frequency between Control and CA12 rats for each morphological class.

As a surrogate marker of neuroinflammation, StainAI computed a microglial activation score using a weighted frequency function (see Methods). Microglial activation scores ranged from 0.11 in MB to 0.28 in SN in the Control slice, and from 0.27 in MB to 0.43 in EC in the CA12 slice (Fig. 3A, B). The microglial activation score provided a convenient, unidimensional metric for comparing microglial activation across multiple locations using standard statistical tools (e.g., ANOVA).

#### Analysis of microglial activation in cortical layers with StainAI

**Fig. 4:**
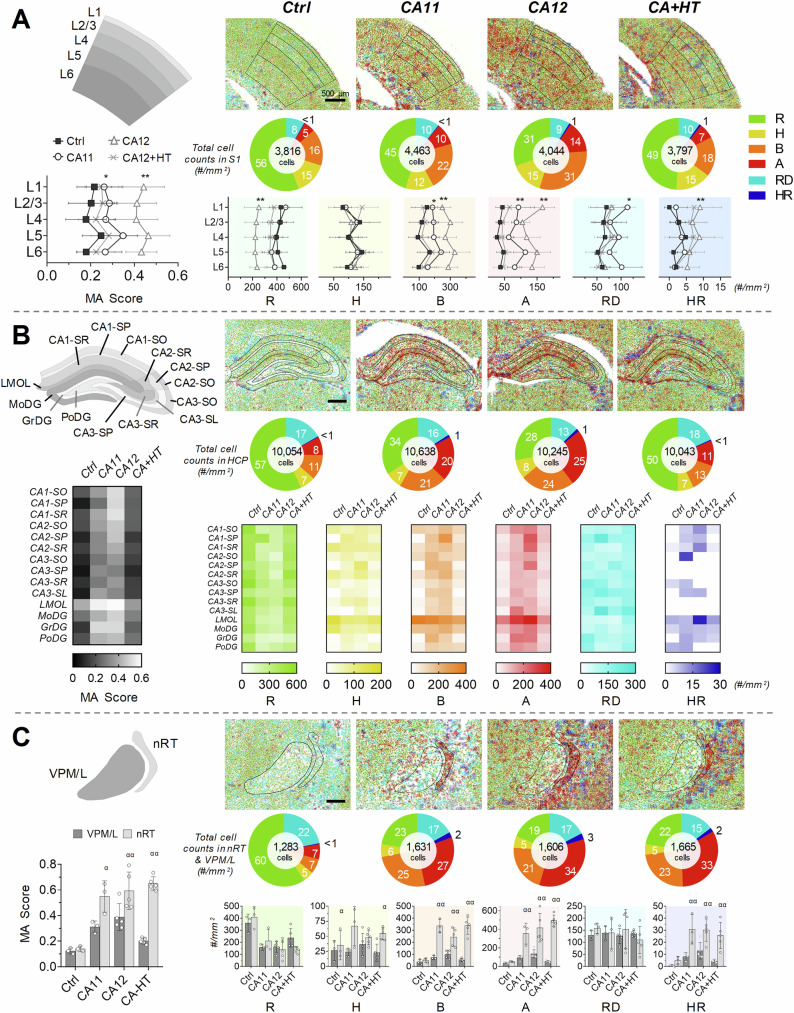
Region-specific microglial activations following cardiac arrest. Morphological analysis of microglia was performed using StainAI for: **A** primary somatosensory cortical layers 1-6 (L1-L6); (**B**) hippocampal sub-regions: cornu ammonis 1, 2, 3 (CA1, CA2, CA3), stratum oriens (SO), stratum pyramidal (SP), stratum radiatum (SR), stratum lucidum (SL), lateral molecular layer of dentate gyrus (LMOL), molecular layer of dentate gyrus (MoDG), granule cell layer of dentate gyrus (GrDG), and polymorphic layer of dentate gyrus (PoDG); (**C**) somatosensory thalamus: thalamic reticular nucleus (nRT) and ventral posterior medial/lateral nucleus (VPM/L). Microglial cell classes were color-coded as follows: ramified (R, green), hypertrophic (H, yellow), bushy (B, orange), ameboid (A, red), rod-shape (RD, cyan), and hypertrophic rod-shaped (HR, blue). Pie charts display the percentage of each cell class relative to total cell count within each region. In the primary somatosensory cortices of control brains, 56% of microglia were ramified, with hypertrophic and bushy cells concentrated in layers 2/3 and 5 (**A**). Cardiac arrest increased total microglial density and activated morphotypes, particularly bushy and ameboid cells, while hypothermia reduced activation, restoring density to control levels. In the hippocampus, microglial density was highest in LMOL and MoDG in controls (**B**). Following cardiac arrest, activated morphotypes increased, particularly in CA12 brains, while hypothermia mitigated this activation, as indicated by heatmaps. In thalamic sub-regions, cardiac arrest led to increased microglial activation in both nRT and VPM/L, whereas hypothermia reduced activation in VPM/L but not in nRT (**C**). Significant regional differences were observed in microglial activation scores. CA11: cardiac arrest for 11 min; CA12: 12 min; CA+HT: 12.5 min with hypothermia treatment, MA: microglial activation. Statistical significance is denoted as *p < 0.05 and **p < 0.01 compared to controls, and ^α^*p *< 0.05 and ^αα^*p *< 0.01 compared to other regions. Data are presented as mean ± standard deviation.

Registration of 2D images to the Waxholm Space Atlas of the Rat Brain enabled quantification of microglial activation scores and microglial classes across the brain (Fig. 4). Figure 4A shows microglial activation scores and the density of each morphological class across six layers of the primary somatosensory cortex as a function of arrest duration and therapeutic hypothermia. Compared to control rats, the microglial activation score in the primary somatosensory cortex across all layers increased by approximately 50% in CA11 rats and 100% in CA12 rats (Supplementary Table 2). Specifically, the scores were 0.20 ± 0.08 in Control rats, 0.29 ± 0.06 in CA11 rats (*p* = 0.042), and 0.43 ± 0.09 in CA12 rats (*p *< 0.0001). Therapeutic hypothermia decreased the microglial activation score in the cortices of CA+HT rats back to baseline (CA+HT: 0.25 ± 0.09, *p* = 0.39). Layer-specific activation patterns revealed heterogeneity in microglial morphology in response to cardiac arrest duration and hypothermia (Fig. 4A). Compared to Control rats, CA11 rats showed a non-significant decrease in the proportion of ramified microglia and mostly non-significant increases in the proportions of other morphologies, except for a slight increase in the proportion of ameboid microglia in layer 5 (Supplementary Table 2). These changes became more pronounced in CA12 rats—the proportion of ramified microglia halved, that of bushy microglia doubled, and that of ameboid microglia tripled compared to Control rats. Hypothermia normalized these distributions in CA+HT rats, although slight increases in hypertrophic and hypertrophic rod-shaped microglia in layer 1 persisted.

#### Analysis of microglial activation in the hippocampus with StainAI

Anatomic segmentation enabled identification of fourteen sub-regions in the hippocampus (Fig. 4B). The detailed segmentation allowed us to calculate microglial densities across sub-regions. The density ranged from a high of 1114 ± 216 cells/mm^2^ in the lacunosum moleculare of the outer layer (LMOL) to a low of 441 ± 202 cells/mm^2^ in the granule cell layer of the dentate gyrus (GrDG). The baseline microglial activation score in Control rats ranged from 0.05 ± 0.01 in CA3 stratum pyramidale (SP) to 0.40 ± 0.16 in LMOL (Supplementary Table 3). The distribution of microglial morphologies also differed among sub-regions in Control rats (Heatmaps in Fig. 4B). Significant differences were observed in the proportion of ramified microglia, which ranged from 71% in CA1-SP to 34% in LMOL (Supplementary Table 4). On the other hand, baseline proportions of hypertrophied and bushy microglia were ten-fold higher in LMOL than in CA1-SP.

Cardiac arrest duration and therapeutic hypothermia exerted sub-region-specific effects on the microglial activation score and microglial morphology (Fig. 4B, Supplementary Tables 3, 4). In CA11 rats, statistically significant increases in microglial activation scores occurred in the CA2-SP, CA3-SP, CA3 stratum lucidum (SL), and in both the GrDG and the polymorphic layer of the dentate gyrus (PoDG). In CA12 rats, statistically significant increases in microglial activation scores occurred in all sub-regions except LMOL. In CA+HT rats, therapeutic hypothermia reduced microglial activation scores back to baseline in all sub-regions, although the scores remained slightly elevated overall (Supplementary Table 3). Morphologically, in CA11 rats, the proportion of ramified microglia decreased in CA2-SP, CA3-SP, GrDG, and PoDG, while the proportion of hypertrophic microglia increased in CA1-SP, and bushy microglia increased in CA1-SP, CA2-SP, and GrDG (Supplementary Table 4). In CA12 rats, the changes were more extensive. The proportion of ramified microglia decreased in ten of the fourteen hippocampal sub-regions. The proportions of bushy microglia increased thirteen-, seven-, and three-fold in CA1-SP, CA2-SP, and CA2-stratum radiatum (SR), respectively; ameboid microglia increased three-, six-, four-, four-, and two-fold in CA1-stratum oriens (SO), CA1-SP, CA1-SR, CA2-SP, and LMOL, respectively; and hypertrophic rod-shaped microglia increased four-fold in LMOL. These changes were quantified using heatmaps in Fig. 4B. As with the microglial activation score, therapeutic hypothermia restored the relative proportion of microglial morphologies back to baseline in all sub-regions. Taken across the entire hippocampus, however, the proportion of ameboid microglia remained increased (Control: 7.7 ± 3.3% vs CA+HT: 11 ± 2.8%, *p *= 0.01). Furthermore, when all “activated” morphological classes (hypertrophic, bushy, ameboid, and hypertrophic rod-shaped) were considered together in CA+HT rats, their proportion exceeded that of Control rats by 19% (*p* < 0.05).

#### Analysis of microglial activation in somatosensory thalamus with StainAI

In the somatosensory thalamus, we focused on two adjacent nuclei known to differ in their response to cardiac arrest-induced injury^29^. The ventral posterior medial/lateral nucleus (VPM/L) serves as the primary relay node for somatosensory information transfer from the brainstem to the cortex and consists of excitatory neurons. The nRT envelops VPM/L in a thin shell of densely interconnected, inhibitory neurons and modulates information processing in VPM/L. VPM/L neurons survived twelve minutes of cardiac arrest, whereas nRT neurons died. Consequently, microglial activation was subtle in VPM/L but markedly pronounced in nRT. Furthermore, therapeutic hypothermia did not affect persistent microglial activation in nRT. We therefore used the VPM/L-nRT circuit as a positive control to quantify the microglial activation score and distribution of microglial morphologies in VPM/L and in nRT as a function of arrest duration and hypothermia.

The VPM/L and nRT microglial activation scores did not differ at baseline in Control rats (Fig. 4C and Supplementary Table 5; nRT: 0.14 ± 0.02 vs VPM/L: 0.12 ± 0.02, *p* = 0.99). In CA11 rats, the nRT microglial activation score exceeded that in VPM/L and quadrupled compared to Control rats (nRT: 0.55 ± 0.12 vs VPM/L: 0.31 ± 0.05, *p* = 0.01; vs Control *p* < 0.01). The VPM/L microglial activation score in CA11 rats did not differ from Control values. In CA12 rats, both VPM/L and nRT microglial activation scores further increased relative to those in Control rats, with the microglial activation score remaining higher in nRT than in VPM/L (nRT: 0.59 ± 0.15 vs VPM/L: 0.39 ± 0.11, *p* = 0.004; nRT or VPM/L vs Control, p’s < 0.01). Hypothermia treatment in CA+HT rats normalized the microglial activation score in VPM/L without affecting the score in nRT (Fig. 4C; nRT: 0.65 ± 0.05 vs VPM/L: 0.20 ± 0.02, *p* < 0.0001; vs Control, *p* < 0.01; VPM/L vs Control, *p* > 0.05). These data show that the microglial activation score accurately identifies the hypothermia-dependent resolution of microglial activation in VPM/L and the hypothermia-resistant persistent microglial activation in nRT.

Baseline morphological class distributions were similar between VPM/L and nRT (Fig. 4C; Supplementary Table 5). In both nuclei of Control rats, ramified and rod-shaped microglia predominated at ~60% and ~20%, respectively. After cardiac arrest, microglial density in these two nuclei increased without an apparent effect of hypothermia, this finding was not observed in the cortex or hippocampus (CA11: 27%, CA12: 25%, and CA+HT: 30%; *p* < 0.01). Microglia in VPM/L and nRT differed in their responses to cardiac arrest and therapeutic hypothermia. In CA11 rats, VPM/L microglia showed only a decrease in the proportion of ramified cells. In contrast, nRT microglia exhibited both a decrease in the proportion of ramified cells and significant, six- to seven-fold increases in the proportions of bushy, ameboid, and hypertrophic rod-shaped microglia (Fig. 4C; Supplementary Table 5). In CA12 rats, with a more severe insult, both VPM/L and nRT showed further increases in the proportions of ameboid and hypertrophic rod-shaped microglia, along with a concomitant decrease in the proportion of ramified cells. Therapeutic hypothermia in CA+HT rats normalized the proportions of morphological classes in VPM/L. However, in nRT, hypothermia had no effect (Fig. 4C). These data provide quantitative evidence that post-arrest therapeutic hypothermia exerts region-specific effects.

### Whole-brain mapping with StainAI

**Fig. 5:**
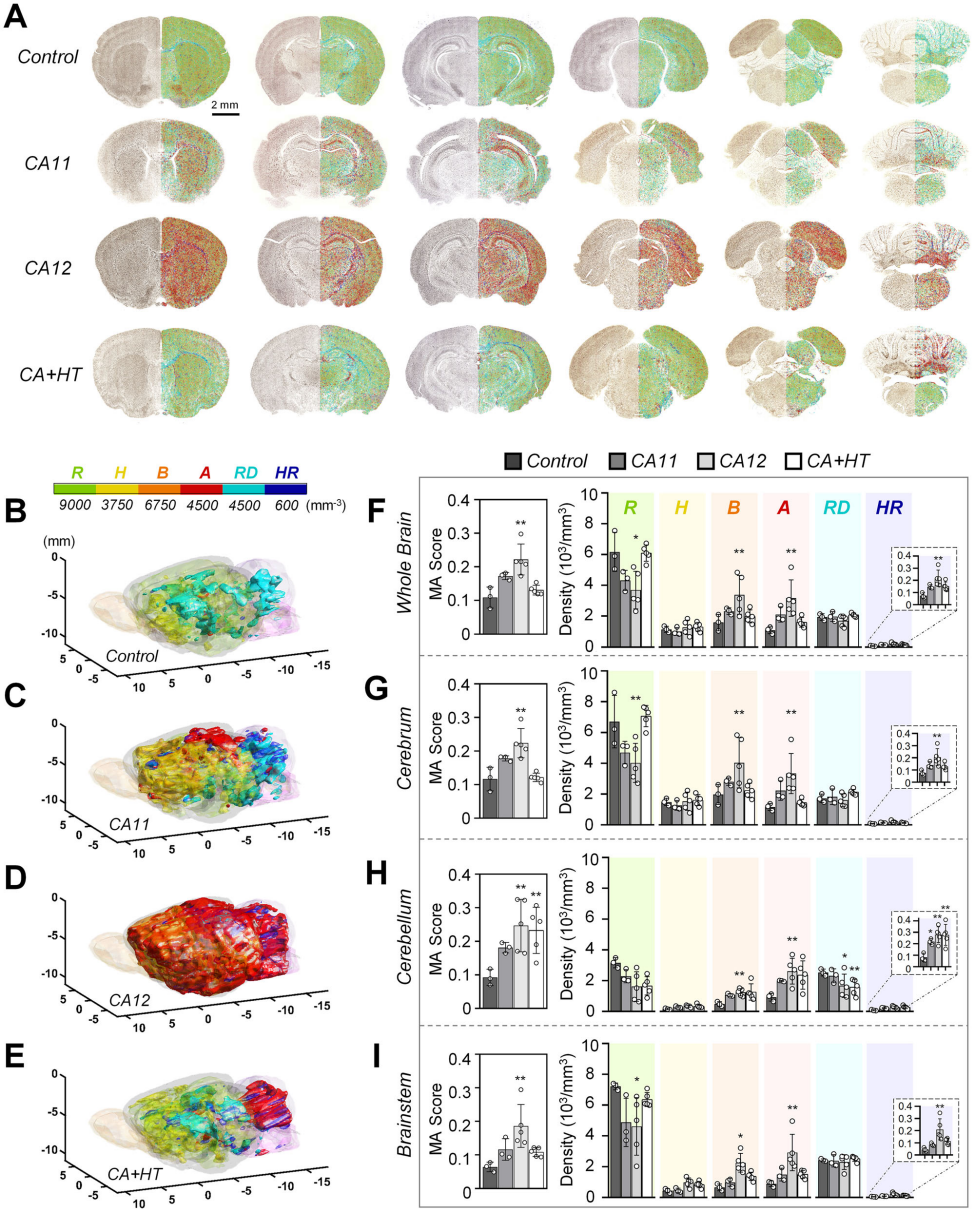
Brain-wide microglial responses to cardiac arrest. **A** Sequential maps illustrate the distribution of microglial morphotypes across the entire brain in each experimental group. **B–E** 3D reconstructions depict whole-brain microglial activation patterns, with contours representing density thresholds for each morphotype (scale bar provided). **F–I** Quantitative analyses reveal microglial activation scores and densities for six morphotypes: ramified (R, green), hypertrophic (H, yellow), bushy (B, orange), ameboid (A, red), rod-shaped (RD, cyan), and hypertrophic rod-shaped (HR, blue). Cardiac arrest resulted in regional increases in microglial activation, particularly in CA12 brains, where ramified microglia decreased to 28%, while activated morphotypes exceeded 60% (**A**, **D**, **F**). Hypertrophic rod-shaped cells significantly increased in the cerebellum (**H**). Hypothermia reduced activation in the cerebrum and brainstem but had limited effects on the cerebellum, as reflected in microglial activation scores (**F**–**I**). CA11: cardiac arrest for 11 minutes; CA12: 12 minutes; CA+HT: 12.5 minutes with hypothermia treatment, MA: microglial activation. * *p *< 0.05, ** *p *< 0.01 compared to controls.

StainAI enabled whole-brain mapping of morphological classes and morphometric features, as well as the construction of 3D iso-surface maps (Fig. 5). StainAI extrapolated morphological class distributions from 2D sequential slices (Fig. 5A) into 3D representations across the encephalon (Fig. 5B–E). Rapid visual inspection identified regions most affected by a given injury. For example, CA11 rats showed the most apparent changes in the anterior and dorsal cerebrum, in the white matter tracts, and in the cerebellum (Fig. 5C). CA12 rats exhibited a widespread shift to ameboid and hypertrophic rod-shaped microglia throughout the entire brain (Fig. 5D). CA+HT rats, on the other hand, clearly showed the region-specific effects of therapeutic hypothermia—the anterior and dorsal cerebrum more closely resembled that of Control and CA11 rats, while the white matter, deep brain nuclei, and especially the cerebellum continued to show extensive injury, similar to CA12 rats (Fig. 5E). StainAI quantified the microglial activation score and volumetric density for each morphological class in arbitrarily large or, with proper anatomic mapping, small anatomical areas (Fig. 5F–I, Supplementary Table 6). Consistent with 3D visualization, the volumetric density of hypertrophic rod-shaped microglia in the cerebellum increased with increasing cardiac arrest duration and did not decrease with therapeutic hypothermia (Control: 82 ± 36, CA11: 218 ± 27; CA12: 280 ± 68; CA+HT: 278 ± 92 cells/mm^3^; *p* < 0.01).

**Fig. 6:**
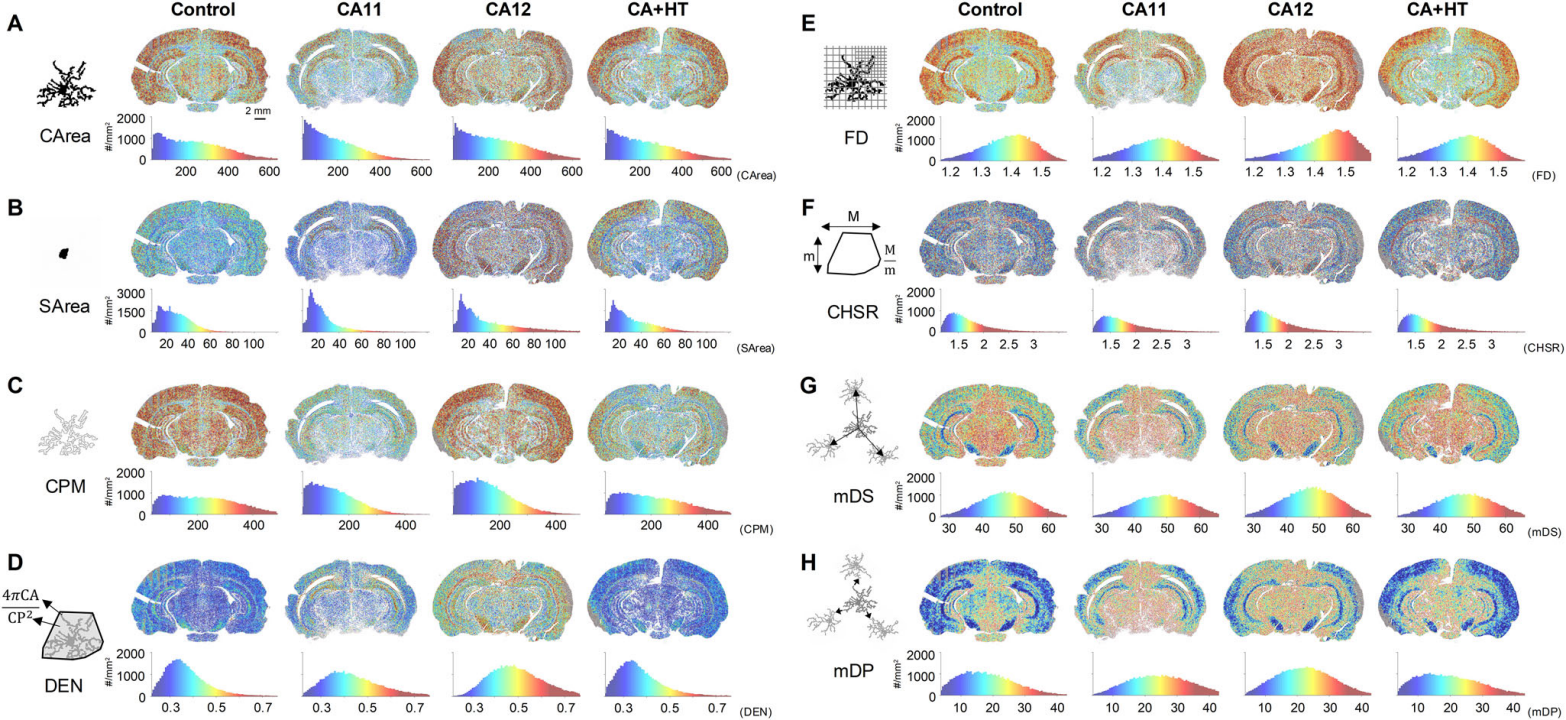
Morphometric parameter maps reveal brain-wide changes in microglial morphology in response to cardiac arrest. Representative maps illustrate: (**A**) cell area (CArea), (**B**) soma area (SArea), (**C**) cell perimeter (CPM), (**D**) density (DEN), (**E**) fractal dimension (FD), (**F**) convex hull span ratio (CHSR), (**G**) mean distance between somas (mDS), and (**H**) mean shortest distance between processes (mDP). Histograms for each parameter display value distributions across the brain. Overall, CA11 brains showed reductions in CArea, SArea, CPM, and FD, while CA12 brains exhibited increases in CArea, DEN, FD, and SArea. CA+HT brains closely resembled controls in CArea, CPM, and DEN but displayed elevated SArea in cortical regions. Maps of CHSR highlight shape changes in white matter, mDS shows decreased soma distances in CA11 and increased distances in CA12, and mDP reveals greater process spacing in CA12, particularly in cortical regions. CA11: cardiac arrest for 11 minutes; CA12: 12 minutes; CA+HT: 12.5 minutes with hypothermia treatment.

The twenty-eight morphometric features characterizing each microglia and its spatial relationship to adjacent microglia (Supplementary Fig. 3) comprised distributions that StainAI calculated from 2D slices or in 3D (Fig. 6). For each feature, both visual and quantitative distributions were computed (Fig. 6A–H), which helped identify regions of interest that required more detailed interrogation. For instance, in coronal sections at the level of the lateral entorhinal cortex, the soma area distribution shifted towards lower values (left) as arrest duration increased (Fig. 6B). Therapeutic hypothermia in CA+HT rats ameliorated the distribution shift but did not fully correct it, particularly in the cortex. Additionally, the visual distribution identified an area of potential interest in the midbrain that also did not respond to hypothermia and may have warranted further study (Fig. 6B, rightmost panel).

### Cross-species application to rhesus macaque brain

**Fig. 7:**
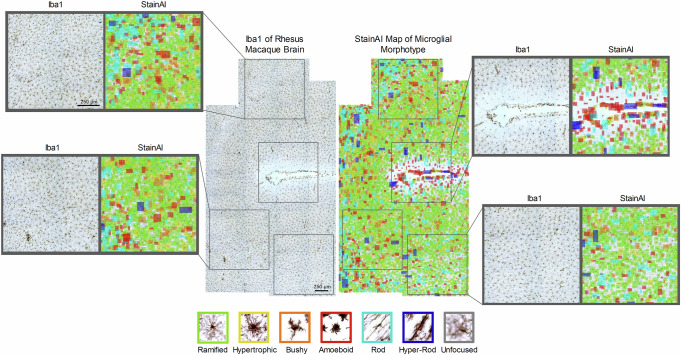
StainAI analysis of pediatric simian immunodeficiency virus (SIV) infection in the rhesus macaque brain. Iba1-stained hippocampal images were acquired 24 h post-infection from a 50 µm brain slice at 20x magnification (0.37 µm/pixel). Multiple images were stitched to cover the entire hippocampus and analyzed using StainAI, which color-coded six distinct microglial morphotypes. Pie charts quantify these morphotypes in specific regions of interest, highlighting rod-shaped microglia surrounding the hippocampus and activated forms within the infected areas. While StainAI has been previously applied to rat brain datasets, this analysis demonstrates its successful extension to rhesus macaque data.

To assess the generalizability of StainAI, it was applied to Iba1-stained images from a SIVmac251-infected rhesus macaque brain. The model successfully identified and quantified six distinct microglial morphotypes in the macaque hippocampus (Fig. 7). Notably, rod-shaped microglia were predominantly located around the hippocampus, alongside various activated morphotypes within the infected regions. Originally developed and successfully applied to rat brain datasets, StainAI has demonstrated its adaptability by effectively analyzing microglial morphology in rhesus macaque tissue.

## Discussion

Histology is one of the standard techniques in most biomedical labs. Without a sensitive tool for reliable image analysis, researchers can struggle to measure visually apparent but unquantifiable data, such as dealing with the heterogeneous microglia in diverse regions. StainAI addresses this gap by transforming IHC images into quantitative maps, enabling easy selection of any region of interest for microglial activation quantification (Fig. 3). The system employs multi-stage, deep learning-based analysis to improve feature extraction from each individual microglia across various backgrounds in commonly used 20x IHC images. The results demonstrated a microglial distribution consistent with previous stereology studies^36^, confirming the expected neuroinflammatory responses in the nRT and CA1 after cardiac arrest^29^. The morphotype and morphometric feature maps further revealed previously unnoticed activation patterns specific to different regions, including cortical layers and hippocampal sub-regions (Fig. 4). They also showed that therapeutic hypothermia reduced microglial activation in the cerebrum and brainstem but not in the cerebellum (Fig. 5). These findings demonstrate that StainAI mapping is proficient at analyzing 2D IHC images of microglia in high-throughput, potentially enhancing its applicability across a wide spectrum of biomedical research and in assessing the efficacy of interventions for various neurological disorders^3,37–42^.

To capture reliable morphological features of microglia, 3D stereology has usually been employed to delineate their delicate cell shapes using high-resolution confocal images^36,43,44^. Computer-assisted programs like MIC-MAC^15^, 3DMorph^16^, and Heindl’s method^14^ have semi-automatically quantified cells within large confocal image stacks. These high-power images (>40x) only capture cells in a narrow FOV, rendering these methods unsuitable for mapping the distribution of microglia throughout the entire brain. Recent advancements in artificial neural network techniques have improved cell quantification^24^. Yet, most published studies have lacked formal validation experiments, limiting their applicability to smaller regions with fewer cells^11,12,27,45^. Single network models like Mask R-CNN^46^ and YOLACT^47^ can analyze full-sized images but struggle to capture nuanced microglial processes (Fig. 2B, Table 2). To comprehensively map microglia throughout the brain, models must distinguish individual microglia from their intertwined processes, while maintaining key cellular details across varying shapes and densities in different brain regions. For instance, in the pons and pituitary gland of a healthy brain, microglial densities were measured at 543 and 578 cells per mm^2^, respectively. Conversely, densely populated regions like the SN and CP had densities nearly doubled to approximately 950 cells per mm^2^, posing a significant challenge for cell separation due to overlapping processes. Histological artifacts and inconsistent image acquisition have further complicated morphological feature extraction, leading researchers to prioritize quantification based on cell number rather than morphology.

The StainAI pipeline employed several effective deep learning-based approaches to address these challenges (Fig. 1). Firstly, it implemented a multi-stage deep learning system that concurrently identified potential cells and achieved high-resolution single-cell segmentation. The system first employed an object detection model to identify microglial cells and define their bounding domains, aiding in the separation of overlapping processes. It then used a segmentation model to capture detailed cell profiles. Secondly, a comprehensive and high-quality ground truth dataset, consisting of 88,897 single-cell masks from various regions and activation states, was employed to ensure robust model training. Additionally, to enhance prediction accuracy and computational efficiency, the pipeline divided large images into smaller sub-image units for analysis (Supplementary Fig. 2). These critical methods enabled precise, stable, and reproducible mapping of microglia on large-sized images, achieving an average DSC of 0.807 in cell segmentation throughout the brain (Table 1).

The morphological changes observed in microglial activation indicate a complex system that, while seemingly random, follows deterministic patterns. This process is tightly regulated by cytokines and chemokines, which influence microglial behavior in both normal and diseased states^48^. The expression of microglial markers has been shown to differ between white and gray matter microglia, with unique patterns found in various brain regions, including the hippocampus, spinal cord, and cerebellum^48,49^. Comparable to earlier reports^50–52^, the StainAI maps showed a higher density of microglia in the SN compared to other gray matter regions such as the cortex and MB (Fig. 3). Over 65% of microglia in cortex and MB exhibited a ramified morphology, whereas only 46% in the SN^53^. The difference may be attributed to elevated neuromelanin levels in SN dopaminergic neurons^36,50^ and other immunoregulatory molecules, such as fractalkine^6^ and CD200^54^. Rod-shaped microglia were common in axon-rich white matter regions, including EC, cerebellar molecular layer, and fimbria area of the hippocampus, reflecting the fibrous microenvironment of major white matter tracts in the brain. Modulating these regional microglial activities has been proposed as a potential therapy for various neurological conditions^55^.

To classify microglia, Fernandez-Arjona et al. introduced a novel categorization system using hierarchical clustering, linear discriminant analysis, and decision trees^4^. Their system analyzed fifteen morphometric parameters to distinguish between normal and infected conditions in rat brains. A later study expanded this system to include eighteen parameters^12^, incorporating additional indices from Sholl analysis^56^ to describe cell geometry of manually selected cells. Microglia were classified into four morphologies (ramified, rod-like, activated, and ameboid), showing higher proportions of activated and rod-like microglia in the hippocampus and cortex after ischemia^12^. The current study classified microglia based on twenty-five morphometric parameters to enhance performance and gain deeper insights. Utilizing these parameters, the C5.0 model classified microglia into six morphotypes, suitable for cells in both gray and white matter, which were validated with high precision (~73%) against manual gold standards (Table 3). Hypertrophic and bushy cells posed the greatest difficulty in accurate classification due to their transitional state between ramified and ameboid morphologies. In addition, large IHC image capture may cause focus quality issues, leading to biases in cell classification. While human experts could still discern cell classes in some blurry images, machine classification struggled due to biased segmentation. Such bias could be mitigated by applying a threshold to focus measure values to identify poorly focused cells (Fig. 2D, E). StainAI created a complete brain map of single-cell morphometry to reveal the changes in microglia across various parameters. Neuroinflammation following cardiac arrest showed clear changes in microglial morphology in many brain regions, including white matter (Fig. 6). After transient cerebral ischemia, increased interactions with pre-synaptic boutons were observed, leading to their disappearance. This suggests that microglia play an active role in synaptic conditions and the remodeling of neuronal circuitry^57^. The two intercellular distancing properties, mean distance between somas and between processes, provided sensitive mappings for soma-process distances, potentially indicating clustering features among individual microglia and their neighboring cells. They may also shed light on the role of microglial processes in synaptic stripping mechanisms^58^. Comprehensive brain mapping techniques like StainAI could offer new insights into microglial heterogeneity, aiding in targeted interventions by integrating transcriptomic data with regional brain maps^50^.

Regional injury despite a seemingly global insult is a hallmark of cardiac arrest-related brain injury^59^. The mechanisms underlying regional vulnerability are not well understood. Traditionally, studies of brain injury in animal models of cardiac arrest have concentrated on a single region, with particular emphasis on the CA1 region of hippocampus^28–30^. Studies of therapeutic interventions, e.g., hypothermia, have also focused on a few regions^60^. The lack of tools to simultaneously and efficiently assess injury and the effects of therapy in multiple areas has hampered our understanding of cardiac arrest-related brain injury^61^. Our data clearly show that microglial activation varied across cortical and hippocampal sub-regions, involved white matter tracts, and affected excitatory and inhibitory thalamic nuclei differently (Fig. 4). Compared to control brains, StainAI estimated a 5–16% increase in microglial density in cardiac arrest brains, driven by proliferation and migration. Early recruitment of microglia occurred in hypoxia-sensitive areas, such as the cortices, hippocampus, and nRT, showing activated morphology and phagocytic activity. After cardiac arrest, microglial activation was evident in the nRT compared to the nearby VPM/L regions of the thalamic circuitry, particularly pronounced in the CA12 brains (Fig. 4C). Interestingly, despite a significant decrease in ramified cells and an increase in bushy and ameboid cells, the number of hypertrophic cells did not notably increase in the injured brains. This suggests that hypertrophic cells may serve as a transitional state between surveillant and activated cell types, maintaining a stable proportion during neuroinflammation.

Multiple preclinical studies have demonstrated that hypothermia improves survival and neurological function in animal models of cardiac arrest^62,63^. However, our findings reveal region-specific limitations in its ability to mitigate microglial activation. In the cortex, hypothermia only partially reduced microglial morphometric changes, suggesting incomplete neuroprotection (Figs. 4, 5). In the nRT, a key regulator of arousal and attention, microglia remained unresponsive to hypothermia, raising concerns about its effectiveness in preserving cognitive function. Similarly, in the cerebellum, now recognized for its role in cognition and motor control, microglial activation persisted despite treatment. These findings emphasize that targeting inflammation in a single region, such as the hippocampus, is insufficient. Effective neuroprotection must address the heterogeneous microglial responses across the brain.

Previously, microglial activation was manually scored using Iba1 staining intensity, cell density, and morphology within a small FOV (0.2–0.5 mm^2^) to assess activation in the peri-contusive cortex after melatonin treatment^64^ and in the hippocampus during zinc-induced neuroinflammation^65^. In the current study, we proposed a universal microglial activation score based on a weighted frequency function of microglial morphotypes. This simplified the microglial activation scoring, with values ranging from 0 (no activation) to 1 (high activation), and intermediate values reflecting varying activation levels throughout the brain. The coefficients in the microglial activation score function (Eq. 1) were derived through empirical regression of millions of microglia, ensuring accurate characterization of activation levels in both gray and white matter regions. In gray matter, R (0) indicates no activation, H (0.33) represents mild activation, B (0.66) reflects moderate activation, and A (1) signifies high activation. In white matter, RD (0) denotes no activation, while HR (0.66) corresponds to moderate activation. These coefficients were calibrated to accurately capture activation levels across the whole brain, improving the scoring system’s precision and reliability in distinguishing microglial activation across different experimental contexts.

Figure 7 demonstrates StainAI’s application to SIV-infected rhesus macaque brain samples, showcasing its versatility in identifying and classifying distinct microglial morphotypes across species. This cross-species capability underscores StainAI’s potential for broader use in neuropathological research, particularly in exploring microglial heterogeneity^32^. Future research may focus on linking these morphological phenotypes to molecular endotypes. Recent studies, such as Jha et al.^66^, have identified distinct microglial clusters in traumatic brain injury using single-cell RNA sequencing. However, while molecular techniques lack spatial context, StainAI allows in-situ analysis of microglial morphology, offering valuable insights into the spatial and temporal relationships between microglial phenotypes and molecular endotypes. Our approach aligns with evolving microglial nomenclature and has the potential to refine classifications by linking morphological features to specific molecular or functional states^67^. Integrating cost-effective morphological phenotyping with molecular endotyping could significantly advance our understanding of microglial function and aid in the development of targeted therapies. Furthermore, StainAI pipeline could also be applied to whole-brain images acquired from light-sheet microscopy with tissue clearing^68^, enabling high-resolution 3D visualization for more precise spatial analysis.

Comparing absolute cell numbers across different studies has been challenging. In the present study, we estimated a higher microglial density compared to earlier reports in adult Long-Evans rat brains^51,52^. This difference was likely due to variations in animal ages, tissue preparation methods, and quantification techniques, particularly adjustments made for tissue shrinkage and threshold settings^69^. The quality of ground truth data was crucial for supervised learning; however, the manual cell labeling process posed challenges, especially when accounting for the varied morphologies of microglia due to their diverse nature. Our manual data indicated that among three experienced neuroscientists, the intra-rater DSC averaged 0.937 ± 0.015, while the inter-rater DSC dropped to 0.750 ± 0.053. The differences in fractal parameters between model predictions and ground truths highlighted the challenges in effectively capturing the complex microglial processes with models (Fig. 2C). In this case, 3D images may be required to accurately depict detailed microglial morphology, especially for rod cells, where sectioning orientation was crucial for cell representation. Cell classification agreement ranged from 0.8 to 0.6 in DSC, both among and between raters. These results revealed the uncertainty in cell labeling, even among well-trained pathologists. The proposed deep learning system yielded comparable results to expert judgment, with a DSC of 0.807 and a classification accuracy of 0.709. Image quality factors, such as focus measure, color tone, dynamic range, background intensity, slide thickness, and staining artifacts, were also important for ensuring reliable cell detection. While decision tree models provided insights into classification-morphometric feature relationships, performance could be improved using alternative models, such as CNN^19^ or parameter reduction via tSNE^70^. Newer methods, including transformer-based approaches^71^, may enhance the ability to capture finer cellular details and resolve transitioning morphotypes.

The performance of morphological analysis is influenced by the quality of input images, which depends on the objective lens used. In this study, a 20x objective lens with a numerical aperture of 0.6 was employed, yielding a spatial resolution of approximately 0.464 μm. Lower numerical aperture values can result in blurrier images, thereby impacting classification accuracy. To mitigate this, Brenner’s focus measure was implemented to exclude poorly focused regions, ensuring reliable analysis across the full-sized whole-brain image. Imaging conditions and post-acquisition processing were also optimized to minimize image quality issues. While StainAI performs well under the current conditions, it is acknowledged that image quality variations, particularly from lower numerical aperture objectives, remain a limitation. Future models should incorporate focus measures into model training, alongside high-quality ground truths, to enable robust analysis of large-scale IHC images. Lastly, the demonstration of the StainAI system on the cardiac arrest model has several limitations that may impact the interpretation of the findings. Differences in sample sizes across groups, driven by practical constraints, may have affected statistical robustness. Similarly, variations in asphyxial insult durations (11 and 12 min for untreated groups, 12.5 min for the hypothermia-treated group), chosen based on typical preclinical responses, could have introduced bias, underscoring the need for standardization in future studies. Although hypothermia treatment inherently could not be blinded, steps were taken to mitigate bias, including randomization of animal assignments and blinding of data analysis. Nonetheless, the absence of full blinding remains a limitation. Additionally, reliance on convenience sampling, dictated by logistical constraints, may have influenced the generalizability of the results. These limitations are presented to enhance transparency and inform improvements in future research efforts.

In conclusion, our study demonstrates the versatility of StainAI in analyzing microglial morphology and its potential for broader applications in neurobiology research. By integrating a robust deep learning system with a comprehensive ground truth database, StainAI eliminates the need for users to create extensive training datasets, as long as their images meet the established quality and format criteria. Although retraining may be required for datasets from species with distinct characteristics, the current platform provides a solid foundation for analyzing standard IHC datasets, advancing microglial research in both healthy and diseased brains.

## Methods

### Rat model of pediatric asphyxial cardiac arrest and resuscitation

Sixteen male and female Long Evans rats (Inotiv, West Lafayette, IL) were induced asphyxial cardiac arrest and resuscitation on postnatal day 17–19^28,29^. Three durations of cardiac arrest were generated: 11 minutes (Group CA11, *n* = 3), 12 minutes (Group CA12, *n* = 5), and 12.5 min cardiac arrest with therapeutic hypothermia (Group CA+HT, *n* = 5). Hypothermia to 34 °C (rectal) was initiated 30 min after resuscitation with a homeothermic temperature control system (Harvard Apparatus, Cambridge, MA) and maintained for 8 h. The rat was then rewarmed to 36 °C over the next 2 h (0.5 °C/h). Three control rats were included for comparison without induction of cardiac arrest (Group Control, *n *= 3). We have complied with all relevant ethical regulations for animal use, and all experimental procedures were performed in accordance with the Institutional Animal Care and Use Committee guidelines at our institutes.

### Immunohistochemistry

24 h after surgery, rats were euthanized and perfused with 4% paraformaldehyde for histology following a published protocol^72^. The entire cohort of brains was processed using MultiBrain technology (NeuroScience Associates, Knoxville, TN), sectioned at 40 μm and stained with primary antibody of Iba1 (Fujifilm Wako Chemicals, Chesterfield, VA, Cat# 019–19741, diluted 1:12,000) and visualized with Ni(II) diaminobenzidine. IHC images were acquired with a pixel resolution of 0.464 μm by a slide scanning MicroBrightField system (MBF Bioscience, Williston, VT) with a 20x (numerical aperture 0.6) objective on an Axioskop microscope (Zeiss Microscopy, Oberkochen, Germany). Images were stitched to cover the whole slide section by the Microlucida software. In each brain, seventeen or eighteen coronal slices were collected at approximately every 1.3 mm in gap covering from the prelimbic cortex to the cerebellum.

### Image pre-processing

To enhance computational efficiency, the full-sized whole-brain image, measuring approximately 12,600 × 7500 μm^2^ with a pixel area of 0.215 μm^2^, was divided into smaller sub-images of 238 × 238 μm^2^ each for deep learning analysis. To ensure accuracy during reassembly, two sets of diagonally shifted sub-image units (unit A and unit B) were created, with a 119 μm gap between them. Unit A identified cells within its boundaries, while Unit B analyzed cells that overlapped with Unit A’s boundary (see Supplementary Information for details).

### Data curation and ground truth

The ground truth database consisted of 88,897 single-cell masks created by experienced neuroscientists following the annotation guideline. A subset of 8695 cells were randomly selected for manual classification into six morphotypes. For model verification, 1290 microglia images from twelve brain regions of a control brain and 1319 images from eleven regions of a cardiac arrest brain were randomly selected for evaluating the accuracy of detection, segmentation, and classification results. These regions included cortex, EC, MB, diencephalon, CA1, CA2, CA3, DG, SN, internal capsule, hindbrain, and pituitary gland.

### Deep learning system

Each sub-image underwent cell detection using YOLOv5, focusing on microglial features at soma and processes. A cell was identified when the class probability of a cell object, *Pr(cell)*, was above 50%. The bounding boxes were then doubled to fully encompass the cell processes and cropped to 119 × 119 μm^2^ for precise UNet segmentation. Each cell received a unique identification and coordinates for subsequent reconstruction. Masks were optimized considering size, proximity, and IoU, defining cell bodies as the largest non-connecting UNet component. Non-connecting fragments smaller than 3.25 μm away from the main body and larger than 107.65 μm^2^ were treated as a separate cell. Overlapping masks (>0.7 IoU) were merged, with fragments assigned to processes based on distance metrics. A total of twenty-eight morphometric parameters were computed from each cell mask. Of these parameters, twenty-five were used to categorize the cell's activation state using C5.0 decision tree classifier to identify key features for classification^73^. The parameters included: six area-related parameters, five perimeter parameters, six span-length related parameters, four span-length ratios, two circularities, and three fractal parameters (Supplementary Fig. 3). Additional two parameters were created to describe intercellular distancing properties of microglia. One difficulty with applying deep learning-based object classification to large histologic sections is varying focus, potentially biasing results. Brenner's focus measure was derived for each cell by their pixel intensity derivatives within an FOV 1.2 times larger than the original bounding box.

Topographic mapping of microglial morphotypes was created using six activation classes and twenty-eight morphometric parameters (Fig. 1C). A color table assigned green, yellow, orange, red, cyan, and blue to represent the ramified, hypertrophic, bushy, ameboid, rod-shaped, and hypertrophic rod-shaped cells, respectively. Cell masks with low focus measure values were grayed out to indicate poor focus quality and excluded from quantification. Morphometric parameter maps were color-coded using a jet colormap according to their respective dynamic ranges. For multi-slice datasets, 3D volume rendering was enabled by identifying slice positions with QuickNII^74^, registering them to the rat brain atlas^75^, and generating 3D iso-surface plots. Cell masks were converted into run-length-encoded and saved with parameters in a JSON file following the COCO dataset format^76^. The deep learning models were trained and evaluated using an NVIDIA GeForce RTX 4090 GPU.

### Model evaluation

The system’s performance was compared to two semantic segmentation and object detection models, Mask R-CNN^46^ and YOLACT^47^ using five-fold cross-validation. mAP_50_ to ground truths was calculated using COCO API^76^. mAP_50_ measures how accurately and consistently the model detects or segments objects in an image, considering both precision (correctness) and recall (completeness) at a specific IoU threshold of 50%. To assess the model's ability in detecting microglia across diverse microenvironments, region-specific microglia detection and segmentation were compared between ground truths and YOLO+UNet predictions. DSC was calculated across eleven brain regions to measure the alignment between segmented regions and true boundaries, with 1 indicating perfect overlap and 0 indicating no overlap. The performance of the C5.0 cell classification model was assessed by comparing its results to ground truths using confusion matrix analysis. Additionally, its performance was compared against various clustering algorithms, including the random forest classifier and linear discriminant analysis. Cohen's kappa was calculated to address the prevalence imbalance of cell morphotypes in the dataset, where ramified cells were usually prevalent, while rod-shaped and hypertrophic rod-shaped cells were less prevalent. Cohen’s kappa ranges from 0 (no agreement) to 1 (perfect agreement), with values between 0.6 and 0.8 indicating substantial agreement. Furthermore, the potential impact of focus quality on bias in cell identification was examined using classification data from 6781 cells.

### Region-specific microglial activation

The trained StainAI models were applied to analyze 288 full-sized Iba1 images of whole brains, focusing on region-specific microglial activations after cardiac arrest. Regions of interest were drawn for quantification in multiple brain regions, including cortex, hippocampus, and thalamus. Based on the Waxholm Space Atlas of the Rat Brain^77,78^, twenty-one brain layers, and sub-regions were delineated, including six cortical layers (L1, 2/3, 4, 5, 6), fourteen hippocampal layers: stratum oriens (SO), stratum pyramidale (SP), stratum radiatum (SR), and stratum lucidum (SL) layers of CA1, CA2, and CA3, lacunosum moleculare of the outer layer (LMOL), molecular layer of the dentate gyrus (MoDG), granule cell layer of the dentate gyrus (GrDG), polymorphic layer of the dentate gyrus (PoDG), and two thalamic regions: nRT, and ventral posterior medial/lateral nucleus (VPM/L). Moreover, the global patterns of microglial activation were quantified and visualized through 3D analysis across whole brain and three anatomical brain regions (cerebrum, brainstem, and cerebellum). Microglial activation score was computed for each region as a surrogate marker for neuroinflammation:

1 $${Microglial\; Activation\; Score}=\frac{0R+0.33H+0.66B+1A+0{RD}+0.66{HR}}{{Total\; Microglia\; Counts}}$$

where *R*, *H*, *B*, *A*, *RD,* and *HR* are density of each morphotype within a given region of interest.

### Analysis of SIV-infected rhesus macaque brain image

To assess the generalizability of the StainAI pipeline, the model was applied to analyze microglial morphology in an archival image of a pediatric rhesus macaque model of oral infection with SIVmac251^31,32^. Iba1-stained hippocampal images were obtained from 50 µm brain sections 24 h post-infection, using the same staining techniques as in the rat study. Imaging was performed at 20x magnification with a resolution of 0.37 µm/pixel. This analysis provided insights into microglial responses in a non-human primate model, demonstrating StainAI’s capability to extend beyond rodent datasets.

### Statistics and Reproducibility

The StainAI-derived matrices (six microglial morphotypes, twenty-eight morphometric parameters, and a microglial activation score) were compared among groups using one-way or two-way ANOVA followed by Tukey's post-hoc multiple comparisons. 2D cell densities were calculated by dividing cell numbers by areas, while 3D densities were computed through volumetric interpolation between slides and normalized to the Waxholm Space Atlas volume^78^. Cells with low focus measure values or a cell area smaller than 30 µm² were excluded from the analysis. Data are presented as the mean ± standard deviation and as percentages relative to the total number of all cell types analyzed. Statistical tests were conducted using Prism 8 (GraphPad Software, La Jolla, CA) and custom MATLAB scripts (MathWorks, Natick, MA), with a significance threshold set at *p* < 0.05. The reproducibility of experiments was confirmed through multiple replicates, with sample sizes and replicate definitions specified for each analysis. Detailed statistical procedures and additional information on experimental reproducibility are provided in the Supplementary Information.

### Reporting summary

Further information on research design is available in the Nature Portfolio Reporting Summary linked to this article.

## Supplementary information

Supplemental Material

Description of Additional Supplementary Materials

Supplementary Data 1

Reporting Summary

## Data Availability

The authors declare that the data supporting the findings of this study are available within the paper and its supplementary information files. Supplementary Data 1 provides detailed raw data. All other data are available from the corresponding author on reasonable request.

## Code availability

The codes used in this study are available on reasonable request from the corresponding author.
