# Supplementary material for: StainAI: quantitative mapping of stained microglia and insights into brain-wide neuroinflammation and therapeutic effects in cardiac arrest
Source: Commun Biol. 2025 Mar 20;8:462. doi: 10.1038/s42003-025-07926-y (PMC11926354; doi:10.1038/s42003-025-07926-y)
Supplement: Supplementary file 1 — Supplemental Material [file 42003_2025_7926_MOESM1_ESM.pdf]

Supplementary Information

StainAI: Quantitative Mapping of Stained Microglia and Insights into Brain-wide Neuroinflammation and Therapeutic Effects in Cardiac Arrest

Chao-Hsiung Hsu[1], Yi-Yu Hsu[1,2], Be-Ming Chang[1], Katherine Raffensperger[3], Micah Kadden[3,4], Hoai T. Ton[3], Essiet-Adidiong Ette[1], Stephen Lin[1], Janiya Brooks[5], Mark W. Burke[5], Yih-Jing Lee[6], Paul C. Wang[1,7], Michael Shoykhet[3,4,8], Tsang-Wei Tu[1,8*]

[1]Molecular Imaging Laboratory, Department of Radiology, Howard University, Washington, DC, USA
[2]Miin Wu School of Computing, National Cheng Kung University, Tainan City, Taiwan
[3]Center for Neuroscience Research, Children's National Research Institute, Washington, DC, USA
[4]Pediatric Critical Care Medicine, Children's National Hospital, Washington, DC, USA
[5]Department of Physiology and Biophysics, Howard University, Washington, DC, USA
[6]School of Medicine, Fu-Jen Catholic University, New Taipei City, Taiwan
[7]Department of Physics, Fu-Jen Catholic University, New Taipei City, Taiwan
[8]Department of Pediatrics, George Washington University School of Medicine and Health Sciences, Washington, DC, USA

*Correspondence should be addressed to

Tsang-Wei Tu, PhD
Associate Professor
Molecular Imaging Laboratory, Department of Radiology, Howard University
2041 Georgia Ave., N.W., Washington, D.C. 20060
Phone: (202) 865-3742;
Fax: (202) 865-3722
E-mail: tsangwei.tu@howard.edu
ORCID ID: 0000-0002-5070-0465

Image data and pre-processing for deep-learning procedures

Microglia were stained for immunohistochemistry (IHC) using ionized calcium-binding adaptor molecule 1 (Iba1, Fujifilm Wako Chemicals, Chesterfield, VA, Cat# 019–19741, diluted 1:12,000) and visualized with Ni(II) diaminobenzidine. The full-sized image was constructed by stitching together multiple snapshots taken at 20x magnification, resulting in a composite image measuring approximately 12,600 × 7,500 μm^2. Each pixel covered an area of 0.215 μm^2, allowing for the complete coverage of the rat brain in coronal view. Images underwent pre-processing to remove obvious staining debris and artifacts, followed by grayscale conversion, intensity normalization, and background removal. The image processing pipeline for microglia image analysis was developed using a combination of in-house MATLAB (Mathworks Inc., Natick, MA) and Python programs. To optimize computational efficiency, each full-sized image was divided into multiple sub-images, each with a reduced field of view (FOV) measuring 238 × 238 μm^2, for deep learning analysis. This partitioning was employed to manage memory requirements effectively and facilitate cell detection and subsequent analysis (Supplementary Fig. 1).

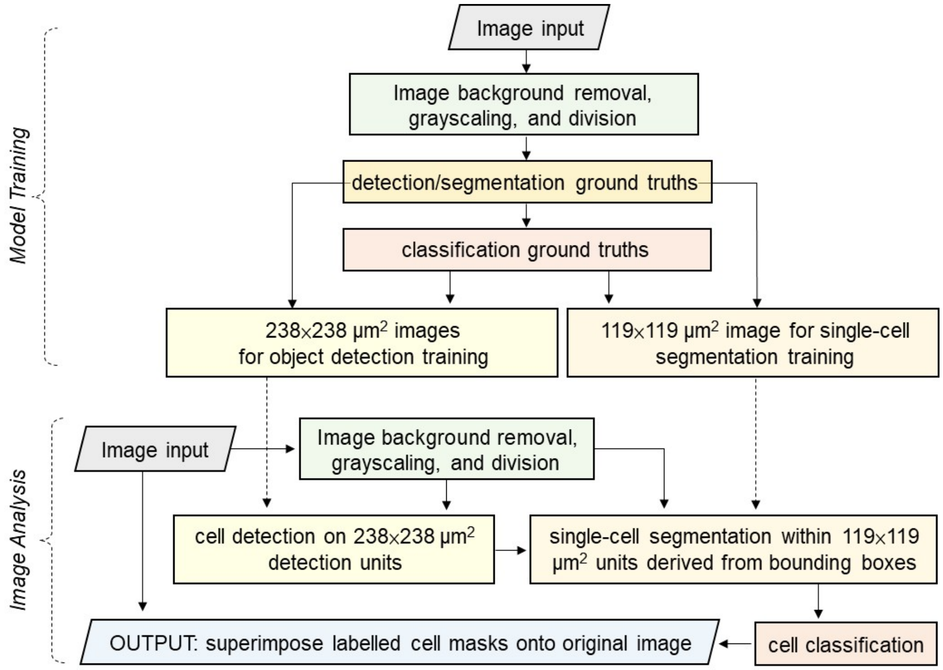

Supplementary Fig. 1 | Flowchart of the multi-stage deep-learning procedures.

Two sets of diagonally shifted sub-image units, labeled Set-A and Set-B, were created for the full-sized image with a 119 μm gap between them. The Set-A units were used to identify cells located entirely within them, excluding those intersecting the boundaries of Set-A units. Conversely, the Set-B units were utilized to identify cells situated within a 10-pixel wide zone adjacent to the boundaries of Set-A units, including cells overlapping with these boundaries (Supplementary Fig. 2).

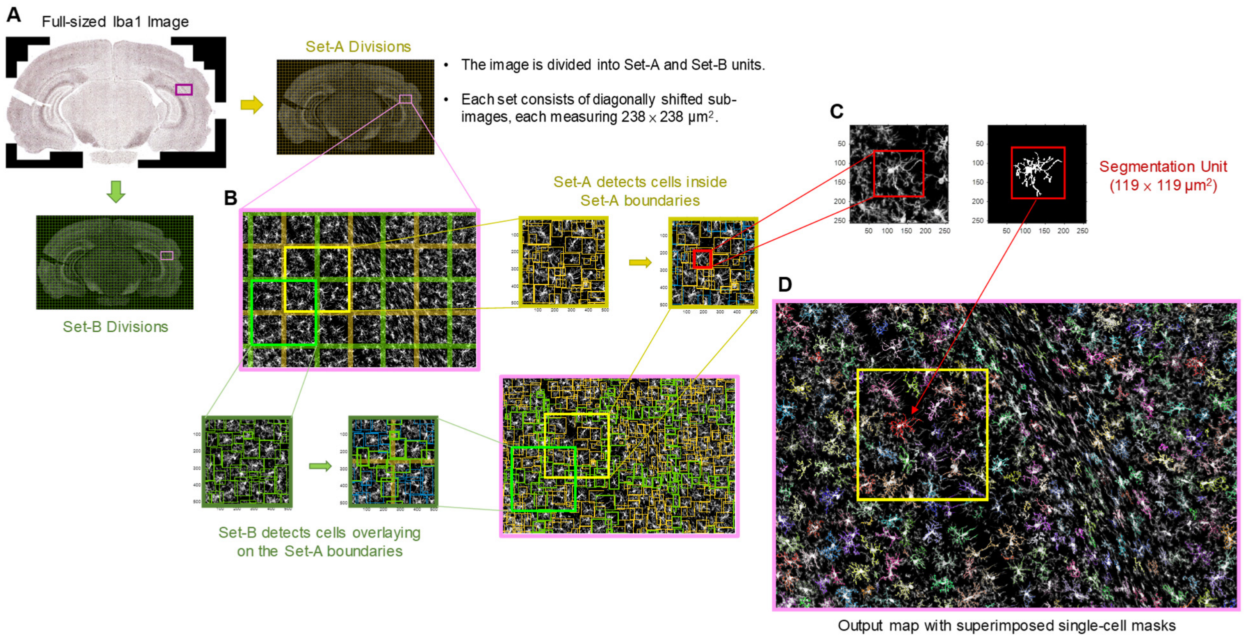

Supplementary Fig. 2 | Diagram illustrating the multi-stage deep-learning procedural pipeline for comprehensive brain image analysis. **A** The full-sized image was initially divided into two sets of sub-images, Set-A and Set-B, with a diagonal shift. **B** Set-A was utilized to detect cells (indicated by yellow bounding boxes) completely within its boundaries, while Set-B was employed to identify cells (indicated by green bounding boxes) within a 10-pixel zone adjacent to the Set-A boundaries. **C** Each bounding box was expanded to ensure full coverage of the extended microglia processes for segmentation. **D** Subsequently, the segmented cell masks were superimposed onto the original image to reconstruct the full-sized cell map.

Deep learning models and data analysis

The multi-stage deep-learning framework consisted of models for cell detection, segmentation and classification. Initially, each sub-image unit (FOV: 238 × 238 μm^2) was

sequentially sent to YOLOv5 for cell detection by pattern recognition of the signature features of microglia, particularly the soma and processes. A cell was identified when the class probability of a cell object, *Pr(cell)*, was above 50%, marking a bounding box that fit most of the cell's extent. These bounding boxes were then enlarged to double their original size to fully cover the extended microglia processes. The bounding boxes, which were twice the original size, were cropped and zero-filled into a FOV of 119 × 119 μm^2 for high-precision segmentation using UNet that consisted of four encoder depth with dice loss function. Each single-cell image was labelled with a unique identification number and coordinates for later reconstruction tasks. After segmentation, individual masks representing single cells were positioned based on their associated coordinates within the IHC image. These masks were processed and optimized using a heuristic rule considering factors such as cell size, proximity, and intersection over union (IoU). Briefly, the cell body was assumed to be the largest non-connecting component in each UNet mask. Fragments smaller than 3.25 μm to the main cell body were retained until the maximum cell size threshold of 970 μm^2 was reached to prevent indefinite growth of cell extent. Non-connecting fragments that were larger than 107.65 μm^2 and 3.25 μm away from the cell body were designated as a separate cell. Two overlapping masks that had an IoU greater than 0.7 were merged and treated as one cell. Finally, overlapping fragments were assigned to the cell processes based on a ranking rule, prioritizing the shortest edge distance, shortest convex hull distance, and shortest maximum intensity distance between the fragment and the cell body.

A total of 28 morphometric parameters were computed from each cell mask (Supplementary Fig. 3). These included 6 area-related parameters, cell area (CArea), convex hull area (CHA), extent (EXT), density (DEN), and soma area (SArea), and (6) the ratio of soma and cell area (rSACA); 4 perimeter-related parameters: (7) cell perimeter (CPM), (8) convex hull perimeter (CHP), (9) soma perimeter (SPM), and (10) the ratio of soma and cell perimeter (rSPCP); 9 shape-related parameters: (11) maximum span across the convex hull (maxSACH), (12) minimum span across the convex hull (minSACH), (13) the major axis length of the ellipse (majAL), (14) the minor axis length of the ellipse (minAL), (15) convex hull span ratio (CHSR), (16) eccentricity (ECC), (17) the diameter of the bounding circle (DBC), (18) the ratio maximum/minimum convex hull radii (rmmCHR), and (19) the mean convex hull radius (mCHR); 6 skeleton parameters: (20) cell circularity (CCIR), (21) convex hull circularity (CHC), (22) fractal dimension (FD), (23) roughness (RG), (24) lacunarity (LC), and (25) standard deviation of lacunarity (stdLC). Two

distancing parameters: (26) mean distance between somas (mDS), and (27) mean shortest distance between processes (mDP), were computed to describe the intercellular distancing properties of microglia. Finally, (28) Brenner's focus measure (FM) was derived for each cell by analyzing their pixel intensity derivatives within an FOV 1.2 times larger than the original bounding box. Out of these parameters, (1)-(25) were utilized to classify the cell's activation state into 6 categories, including ramified (R), hypertrophic (H), bushy (B), amoeboid (A), rod (RD), and hyper-rod (HR) cells. These parameters were applied to classification models, aiming to understand the crucial features involved in classifying the heterogeneous microglial cells.

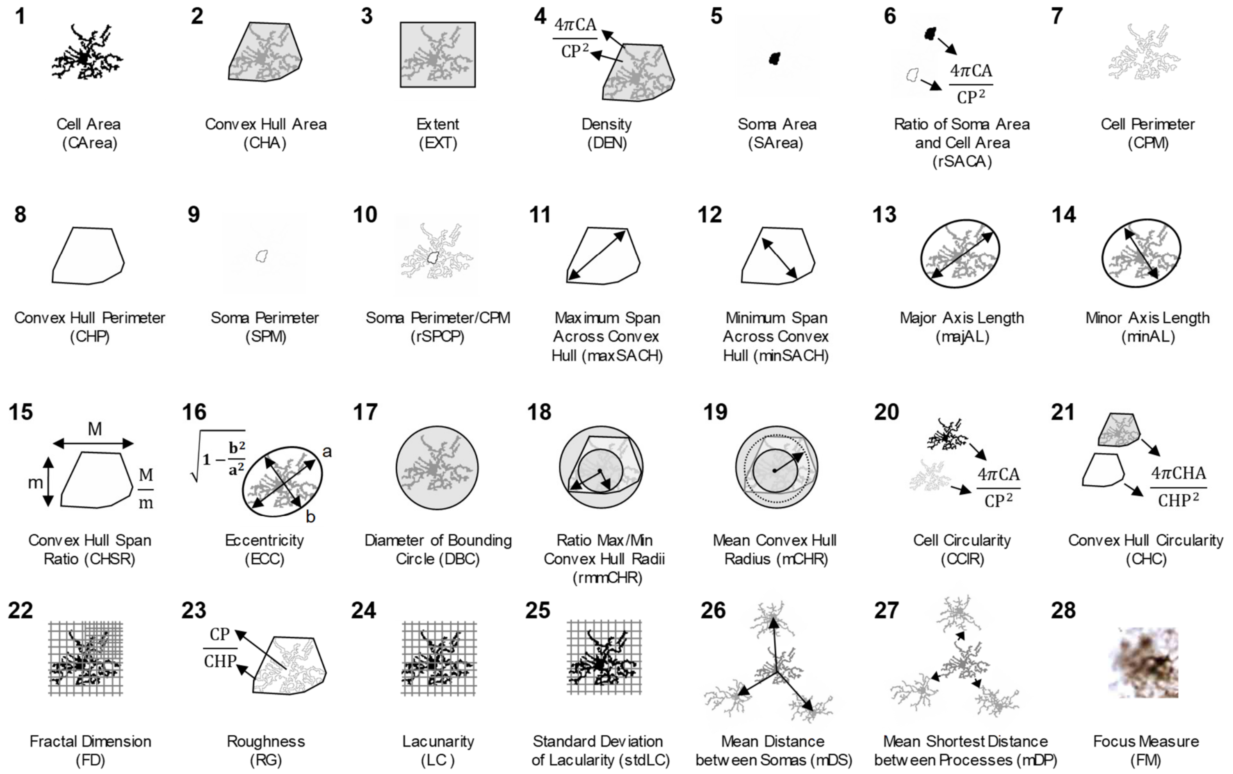

Supplementary Fig. 3 | Morphometric parameters of microglia derived in StainAI. These parameters describe various morphological features including area (1-6), perimeter (7-10), shape (11-19), skeleton (20-25), distance between cells (26, 27), and image blurriness (28).

Evaluation of classification model

The C5.0 cell classification results were evaluated by conducting a comparative analysis of the accuracy and F1 score to multiple clustering algorithms, including the newer meta-

estimators, such as random forest classifier, and linear discriminant analysis, that are commonly used to extract informative patterns from deep learning results (Supplementary Table 1). 1,290 microglia images from 12 brain regions of a control brain and 1,319 images from 11 regions of a cardiac arrested brain were randomly selected for evaluations. These regions included the cortex, external capsule, midbrain, diencephalon, cornu ammonis 1, 2, 3, dentate gyrus, substantia nigra, internal capsule, hindbrain, and pituitary gland. All computational methods underwent validation by comparing them to expert-defined gold-standard ground truths.

Supplementary Table 1 | Accuracy and F1 score of the classification models assessed for microglia classification, compared to a human-curated ground truth dataset comprising 2,609 cells. Models were trained using morphometric parameters (1) to (25) detailed in Supplementary Fig. 3.

Model	Accuracy	F1 Score
C5.0 Decision Tree	0.721	0.719
SVC	0.710	0.704
LGBM Classifier	0.707	0.706
Logistic Regression	0.707	0.703
Linear Discriminant Analysis	0.700	0.697
Random Forest Classifier	0.699	0.690
XGB Classifier	0.696	0.689
Calibrated Classifier CV	0.693	0.675
Linear SVC	0.688	0.668
Extra Trees Classifier	0.685	0.675

Image reconstruction and morphological mapping

The topographic mapping of microglial morphology was reconstructed from 6 activation morphotypes and 28 morphometric parameters. For activation classes, a color table was used, assigning green, yellow, orange, red, cyan and blue to represent the morphotype R, H, B, A, RD and HR, respectively. Cell masks with low FM values were grayed out to indicate poor focus quality and were subsequently excluded from the quantification process. The morphometric parameter maps were color-coded using a jet colormap with defined display ranges. For multi-slice dataset, 3D volume rendering was enabled through the following pipeline. The position of each 2D slice was initially identified using the QuickNII tool, registered to the rat brain atlas. 3D iso-surface plots were

generated by interpolating the data points between the 2D slices, which involved calculating the cell density per mm^3 within each sub-image region (~0.12 um^2). All cell masks were converted into run-length-encoded and saved with morphological parameters in the JSON file following the format of COCO dataset.

Quantification of region-specific microglial activations

The trained StainAI models were used for quantitative histology to examine 288 full-sized Iba1 images from 16 rat brains. Three durations of cardiac arrest were generated: 11 minutes (Group CA11, n = 3), 12 minutes (Group CA12, n = 5), and 12.5 minutes asphyxia cardiac arrest + hypothermia treatment (Group CA+HT, n = 5). Three control rats were included for comparison without induction of cardiac arrest (Group Control, n=3). A morphotype map and 28 morphometry maps of microglia were computed for every Iba1 image using the pipeline illustrated in Fig 1 of the main text and detailed in Supplementary Figure 3. These maps were used for regions of interest (ROIs) analysis to quantify the region-specific microglial activation in the primary somatosensory cortex (S1), hippocampus (HPC) and thalamus (THAL). According to the Waxholm Space Atlas of the Rat Brain, 21 sub-regions were delineated, including 6 S1 layers (L1, 2/3, 4, 5, 6), 14 hippocampal sub-regions: stratum oriens (SO), stratum pyramidale (SP), stratum radiatum (SR), and stratum lucidum (SL) layers of cornu ammonis 1, 2, 3 (CA1, CA2, CA3), lacunosum moleculare of the outer layer (LMOL), molecular layer of the dentate gyrus (MoDG), granule cell layer of the dentate gyrus (GrDG), polymorphic layer of the dentate gyrus (PoDG), and 2 thalamic regions: thalamic reticular nucleus (nRT), and ventral postero-medial & lateral nucleus (VPM/L). Furthermore, the activation of microglia was visualized and quantified through 3D analysis across the entire brain (whole brain), and three major brain divisions – cerebrum, brainstem, and cerebellum.

Statistical analysis

The StainAI-derived matrices (6 microglial morphotypes, 28 morphometric parameters, and microglial activation score) were compared across the Control, CA11, CA12, and CA+HT groups using one-way or two-way ANOVA followed by Tukey's post-hoc multiple comparisons. 2D cell densities were calculated by dividing cell numbers by areas, while 3D densities were computed through volumetric interpolation between slides and normalized to the Waxholm Space Atlas volume and excluded the cells with low FM values or CArea smaller than 30 μm^2. Detailed statistical data are provided for somatosensory cortex (Supplementary Table 2), hippocampus (Supplementary Tables 3 and 4), thalamic regions (Supplementary Table 5), and brain divisions

(Supplementary Table 6). Data were presented as mean ± standard deviation, and as percentages relative to the total numbers of all cell types. All statistical tests were performed using Prism 8 (GraphPad Software, La Jolla, CA) and custom MATLAB (MathWorks, Natick, MA) scripts with a significance level of $p < 0.05$.

Supplementary Table 2 | Layer-specific microglial activations in the somatosensory cortical layers following cardiac arrest and hypothermia treatment. Cell density was reported in counts per mm^2.

	Layer	Control (n=3)		CA11 (n=3)		CA12 (n=5)		CA+HT (n=5)	
**MA Score**	L1	0.22 ± 0.08		0.26 ± 0.08		0.44 ± 0.10**		0.25 ± 0.08	
	L2/3	0.20 ± 0.11		0.29 ± 0.03		0.41 ± 0.09**		0.26 ± 0.07	
	L4	0.18 ± 0.09		0.27 ± 0.05		0.41 ± 0.10**		0.22 ± 0.08	
	L5	0.25 ± 0.09		0.35 ± 0.07		0.46 ± 0.10**		0.28 ± 0.08	
	L6	0.18 ± 0.04		0.27 ± 0.08		0.43 ± 0.07***		0.23 ± 0.09	
		count	***%***	***count***	***%***	***count***	***%***	***count***	***%***
**R**	L1	431 ± 69$^{\varepsilon}$	*(56)*	472 ± 135	*(49)*	251 ± 120*	*(29)*	377 ± 40	*(47)*
	L2/3	429 ± 107	*(55)*	423 ± 55	*(46)*	230 ± 93**	*(29)*	349 ± 69	*(47)*
	L4	398 ± 93	*(58)*	391 ± 58	*(46)*	222 ± 85*	*(28)*	346 ± 57	*(51)*
	L5	407 ± 77	*(51)*	357 ± 75	*(39)*	221 ± 78**	*(25)*	342 ± 69	*(44)*
	L6	457 ± 21$^{\alpha}$	*(61)*	381 ± 72	*(46)*	237 ± 60**	*(28)*	382 ± 60	*(54)*
**H**	L1	80 ± 29	*(10)*	90 ± 6	*(10)*	81 ± 48	*(9)*	145 ± 55	*(18)*
	L2/3	137 ± 68	*(17)*	128 ± 14	*(14)*	109 ± 35	*(14)*	128 ± 43	*(17)*
	L4	99 ± 62	*(14)*	101 ± 31	*(12)*	106 ± 40	*(13)*	98 ± 42	*(14)*
	L5	144 ± 40	*(18)*	129 ± 39	*(14)*	136 ± 33	*(15)*	151 ± 60	*(19)*
	L6	95 ± 45	*(12)*	107 ± 36	*(13)*	122 ± 28	*(14)*	112 ± 53	*(15)*
**B**	L1	151 ± 102	*(19)*	185 ± 33	*(20)*	248 ± 55	*(31)*	127 ± 50	*(16)*
	L2/3	117 ± 82	*(14)*	193 ± 33	*(21)*	300 ± 80**	*(34)*	139 ± 44	*(20)*
	L4	92 ± 60	*(13)*	194 ± 34	*(23)*	268 ± 73**	*(31)*	115 ± 45	*(16)*
	L5	167 ± 79	*(20)*	241 ± 53	*(26)*	329 ± 96**	*(36)*	184 ± 79	*(22)*
	L6	98 ± 42	*(13)*	155 ± 59	*(19)*	287 ± 62**	*(33)*	130 ± 70	*(17)*
**A**	L1	44 ± 21	*(6)*	93 ± 50	*(10)*	166 ± 38$^{***\beta}$	*(20)*	65 ± 29	*(8)*
	L2/3	36 ± 13	*(5)*	86 ± 15	*(10)*	105 ± 23$^{*\alpha}$	*(14)*	44 ± 17	*(6)*
	L4	27 ± 10	*(4)*	63 ± 16	*(7)*	118 ± 41***	*(15)*	42 ± 21	*(6)*
	L5	46 ± 21	*(6)*	114 ± 37*	*(12)*	150 ± 42***	*(17)*	54 ± 17	*(7)*
	L6	37 ± 7	*(5)*	81 ± 22	*(10)*	134 ± 38***	*(16)*	46 ± 30	*(6)*
**RD**	L1	70 ± 33	*(9)*	113 ± 7	*(12)*	81 ± 31	*(10)*	81 ± 49	*(10)*
	L2/3	64 ± 26	*(8)*	77 ± 34	*(9)*	63 ± 16	*(9)*	71 ± 30	*(9)*
	L4	67 ± 15	*(10)*	95 ± 17	*(11)*	86 ± 15	*(12)*	80 ± 35	*(11)*
	L5	52 ± 7	*(6)*	76 ± 14	*(8)*	50 ± 23	*(6)*	59 ± 20	*(7)*
	L6	61 ± 16	*(8)*	101 ± 42	*(12)*	67 ± 10	*(8)*	53 ± 17	*(8)*
**HR**	L1	0 ± 0	*(0)*	2 ± 3	*(0)*	9 ± 6*	*(1)*	7 ± 6	*(1)*
	L2/3	3 ± 3	*(0)*	3 ± 2	*(0)*	7 ± 4	*(1)*	6 ± 4	*(1)*
	L4	5 ± 2	*(1)*	4 ± 3	*(0)*	9 ± 4	*(1)*	7 ± 8	*(1)*
	L5	1 ± 1	*(0)*	4 ± 2	*(0)*	6 ± 4	*(1)*	6 ± 2	*(1)*
	L6	2 ± 2	*(0)*	2 ± 1	*(0)*	5 ± 4	*(1)*	2 ± 1	*(0)*

MA: microglial activation, **R**: ramified, **H**: hypertrophic, **B**: bushy, **A**: ameoboid, **RD**: rod, **HR**: hyper-rod, **CA**: cardia arrest, **CA11**: 11 mins cardiac arrest, **CA12**: 12 mins cardiac arrest, **CA+HT**: Hypothermia treatment after 12.5 mins cardiac arrest, **L**: layer; *$p < 0.05$, **$p < 0.01$, ***$p < 0.001$: vs. control; $p < 0.05$: $^{\alpha}$vs L1, $^{\beta}$vs L2/3, $^{\gamma}$vs L4, $^{\delta}$vs L5, $^{\varepsilon}$vs L6; all data are reported as mean ± SD.

Supplementary Table 3 | Sub-region-specific microglial activations in hippocampus following cardiac arrest and hypothermia treatment (measured by microglial activation score).

	Layer	Control (n=3)	CA11 (n=3)	CA12 (n=5)	CA+HT (n=5)
	CA1-SO	0.21 ± 0.06	0.36 ± 0.15	$0.51 \pm 0.16^{**}$	0.24 ± 0.07
	CA1-SP	$0.07 \pm 0.05^{\nu}$	$0.26 \pm 0.03^{\nu}$	$0.50 \pm 0.19^{**}$	0.22 ± 0.11
	CA1-SR	0.19 ± 0.08	0.39 ± 0.10	$0.53 \pm 0.14^{**\theta}$	0.23 ± 0.05
	CA2-SO	0.18 ± 0.07	0.37 ± 0.09	$0.46 \pm 0.18^{**}$	0.22 ± 0.12
	CA2-SP	$0.09 \pm 0.08^{\nu}$	$0.32 \pm 0.02^{*}$	$0.46 \pm 0.11^{**}$	$0.14 \pm 0.09^{\nu}$
	CA2-SR	0.15 ± 0.10	0.32 ± 0.09	$0.41 \pm 0.13^{**}$	0.18 ± 0.01
**MA Score**	CA3-SO	$0.09 \pm 0.02^{\nu}$	$0.27 \pm 0.01^{\nu}$	$0.35 \pm 0.10^{**\nu}$	0.15 ± 0.08
	CA3-SP	$0.05 \pm 0.01^{\nu}$	$0.32 \pm 0.06^{**}$	$0.30 \pm 0.16^{**\gamma\nu}$	$0.10 \pm 0.02^{\nu}$
	CA3-SR	$0.12 \pm 0.05^{\nu}$	0.28 ± 0.06	$0.35 \pm 0.15^{**\nu}$	0.15 ± 0.04
	CA3-SL	$0.07 \pm 0.06^{\nu}$	$0.37 \pm 0.04^{**}$	$0.32 \pm 0.15^{**\nu}$	0.15 ± 0.05
	LMOL	$0.40 \pm 0.16^{\beta\varepsilon\eta\theta\lambda\mu}$	$0.56 \pm 0.10^{\beta\eta}$	$0.59 \pm 0.14^{\eta\theta\lambda\mu}$	$0.36 \pm 0.07^{\varepsilon\theta}$
	MoDG	0.27 ± 0.09	0.41 ± 0.04	$0.49 \pm 0.10^{*}$	0.30 ± 0.06
	GrDG	0.13 ± 0.06	$0.51 \pm 0.02^{**}$	$0.51 \pm 0.14^{**}$	0.22 ± 0.12
	PoDG	0.19 ± 0.04	$0.42 \pm 0.12^{*}$	$0.44 \pm 0.11^{**}$	0.27 ± 0.09

MA: microglial activation, **CA**: cardia arrest, **CA11**: 11 mins cardiac arrest, **CA12**: 12 mins cardiac arrest, **CA+HT**: Hypothermia treatment after 12.5 mins cardiac arrest, **CA**: cornu ammonis, **SO**: stratum oriens, **SP**: stratum pyramidale, **SR**: stratum radiatum, **SL**: stratum lucidum, **LMOL**: lacunosum moleculare of the outer layer, **MoDG**: molecular layer of the dentate gyrus, **GrDG**: granule cell layer of the dentate gyrus, **PoDG**: polymorphic layer of the dentate gyrus; *p < 0.05, **p < 0.01: vs. control; $p<0.05$: $^{\alpha}$vs CA1-SO, $^{\beta}$vs CA1-SP, $^{\gamma}$vs CA1-SR, $^{\delta}$vs CA2-SO, $^{\varepsilon}$vs CA2-SP, $^{\zeta}$vs CA2-SR, $^{\eta}$vs CA3-SO, $^{\theta}$vs CA3-SP, $^{\lambda}$vs CA3-SR, $^{\mu}$vs CA3-SL, $^{\nu}$vs LMOL, $^{\xi}$vs MoDG, $^{\pi}$vs GrDG, $^{\rho}$vs PoDG; all data are reported as mean ± SD.

Supplementary Table 4 | Region-specific microglial activations in hippocampus following cardiac arrest and hypothermia (by microglial morphotypes). Cell density was reported in counts per mm^2.

	Layer	Control (n=3) count	%	CA11 (n=3) count	%	CA12 (n=5) count	%	CA+HT (n=5) count	%
**R**	CA1-SO	487 ± 52$^{\pi}$	*(57)*	238 ± 61	*(37)*	176 ± 97**	*(23)*	401 ± 51$^{\pi}$	*(50)*
	CA1-SP	349 ± 106	*(64)*	425 ± 69$^{\pi\rho}$	*(42)*	217 ± 93**	*(25)*	335 ± 52	*(48)*
	CA1-SR	455 ± 67	*(58)*	235 ± 79	*(35)*	169 ± 83**	*(24)*	414 ± 44$^{\pi}$	*(52)*
	CA2-SO	552 ± 226$^{\theta\pi}$	*(63)*	225 ± 56	*(32)*	231 ± 203**	*(30)*	403 ± 49$^{\pi}$	*(53)*
	CA2-SP	361 ± 81	*(71)*	322 ± 99*	*(40)*	190 ± 88**	*(23)*	363 ± 90	*(61)*
	CA2-SR	476 ± 87$^{\pi}$	*(64)*	290 ± 69	*(41)*	178 ± 111**	*(26)*	461 ± 34$^{\pi}$	*(59)*
	CA3-SO	496 ± 83$^{\pi}$	*(64)*	292 ± 63	*(43)*	239 ± 100**	*(37)*	376 ± 84$^{\pi}$	*(59)*
	CA3-SP	259 ± 119$^{\delta}$	*(56)*	260 ± 43**	*(34)*	238 ± 63**	*(37)*	274 ± 68	*(55)*
	CA3-SR	485 ± 45$^{\pi}$	*(66)*	314 ± 64	*(45)*	253 ± 113	*(37)*	444 ± 44$^{\pi}$	*(60)*
	CA3-SL	419 ± 109	*(70)*	259 ± 16	*(33)*	244 ± 95	*(38)*	353 ± 64	*(51)*
	LMOL	351 ± 125	*(34)*	206 ± 73	*(20)*	191 ± 119	*(19)*	387 ± 81$^{\pi}$	*(37)*
	MoDG	408 ± 77	*(49)*	248 ± 43	*(32)*	198 ± 73*	*(25)*	359 ± 66$^{\pi}$	*(45)*
	GrDG	219 ± 130$^{\alpha\delta\zeta\eta\lambda}$	*(46)*	149 ± 30$^{**\beta}$	*(21)*	117 ± 58**	*(21)*	164 ± 53$^{\alpha\gamma\delta\zeta\eta\lambda\nu\xi}$	*(43)*
	PoDG	381 ± 164	*(53)*	172 ± 19$^{*\beta}$	*(31)*	188 ± 97**	*(31)*	282 ± 80	*(41)*
**H**	CA1-SO	83 ± 50	*(9)*	47 ± 15	*(7)*	63 ± 40	*(8)*	68 ± 22	*(8)*
	CA1-SP	4 ± 7$^{\nu\xi}$	*(1)*	89 ± 6*	*(9)*	79 ± 42*	*(9)*	48 ± 23	*(7)*
	CA1-SR	79 ± 62	*(9)*	66 ± 27	*(10)*	67 ± 38	*(9)*	77 ± 21	*(9)*
	CA2-SO	45 ± 31$^{\nu}$	*(5)*	62 ± 36	*(9)*	60 ± 65	*(10)*	61 ± 25	*(8)*
	CA2-SP	30 ± 52$^{\nu}$	*(4)*	43 ± 8	*(5)*	83 ± 77	*(10)*	33 ± 47	*(5)*
	CA2-SR	62 ± 54$^{\nu}$	*(7)*	61 ± 13	*(9)*	76 ± 38	*(11)*	47 ± 41	*(6)*
	CA3-SO	31 ± 27$^{\nu}$	*(4)*	24 ± 18	*(4)*	35 ± 5	*(6)*	29 ± 21$^{\nu}$	*(4)*
	CA3-SP	6 ± 10$^{\nu\xi}$	*(1)*	41 ± 7	*(5)*	49 ± 32	*(7)*	10 ± 7$^{\nu\xi}$	*(2)*
	CA3-SR	32 ± 20$^{\nu}$	*(4)*	71 ± 19	*(10)*	72 ± 28	*(10)*	39 ± 19$^{\nu}$	*(5)*
	CA3-SL	10 ± 9$^{\nu\xi}$	*(2)*	26 ± 46	*(4)*	50 ± 42	*(7)*	14 ± 9$^{\nu\xi}$	*(2)*
	LMOL	157 ± 59$^{\beta\delta\varepsilon\zeta\eta\theta\lambda\mu\pi\rho}$	*(14)*	101 ± 38	*(10)*	94 ± 52	*(9)*	136 ± 32$^{\eta\theta\lambda\mu\pi\rho}$	*(13)*
	MoDG	111 ± 44$^{\beta\theta\mu\pi}$	*(13)*	72 ± 22	*(9)*	81 ± 39	*(10)*	88 ± 19$^{\theta\mu}$	*(11)*
	GrDG	10 ± 5$^{\nu\xi}$	*(2)*	51 ± 10	*(7)*	32 ± 10	*(5)*	18 ± 31$^{\nu}$	*(3)*
	PoDG	22 ± 24$^{\nu}$	*(3)*	32 ± 16	*(5)*	26 ± 30	*(4)*	45 ± 15$^{\nu}$	*(7)*
**B**	CA1-SO	116 ± 75$^{\nu}$	*(13)*	140 ± 96$^{\nu}$	*(20)*	188 ± 75$^{\nu}$	*(24)*	111 ± 35$^{\nu}$	*(14)*
	CA1-SP	15 ± 18$^{\nu}$	*(2)*	163 ± 68.71*	*(17)*	260 ± 86**	*(26)*	121 ± 107	*(15)*
	CA1-SR	97 ± 65$^{\nu}$	*(11)*	164 ± 50	*(25)*	187 ± 51	*(26)*	123 ± 55	*(15)*
	CA2-SO	106 ± 67$^{\nu}$	*(12)*	182 ± 130	*(24)*	152 ± 59	*(23)*	113 ± 79	*(14)*
	CA2-SP	22 ± 39$^{\nu}$	*(3)*	209 ± 38**	*(26)*	176 ± 100**	*(21)*	38 ± 44$^{\nu}$	*(6)*
	CA2-SR	85 ± 55$^{\nu}$	*(11)*	161 ± 80	*(23)*	220 ± 62*	*(34)*	84 ± 30$^{\nu}$	*(11)*
	CA3-SO	41 ± 25$^{\nu}$	*(5)*	91 ± 33$^{\nu}$	*(13)*	127 ± 37$^{\nu}$	*(21)*	56 ± 39$^{\nu}$	*(9)*
	CA3-SP	7 ± 12$^{\nu}$	*(1)*	138 ± 21$^{\nu}$	*(18)*	123 ± 46$^{\nu}$	*(18)*	13 ± 9$^{\nu}$	*(3)*
	CA3-SR	38 ± 31$^{\nu}$	*(5)*	109 ± 26$^{\nu}$	*(15)*	133 ± 56$^{\nu}$	*(19)*	54 ± 25$^{\nu}$	*(7)*
	CA3-SL	15 ± 13$^{\nu}$	*(2)*	94 ± 47$^{\nu}$	*(12)*	129 ± 69$^{\nu}$	*(18)*	36 ± 25$^{\nu}$	*(5)*
	LMOL	344 ± 231$^{\alpha\beta\gamma\delta\varepsilon\zeta\eta\theta\lambda\mu\xi\pi\rho}$	*(29)*	328 ± 54$^{\alpha\eta\theta\lambda\mu\pi\rho}$	*(32)*	310 ± 94$^{\alpha\eta\theta\lambda\mu\pi\rho}$	*(30)*	249 ± 100$^{\alpha\varepsilon\zeta\eta\theta\lambda\mu\pi\rho}$	*(23)*
	MoDG	161 ± 86$^{\nu}$	*(18)*	185 ± 44	*(24)*	227 ± 48	*(29)*	152 ± 32$^{\theta}$	*(19)*
	GrDG	13 ± 14$^{\nu}$	*(2)*	204 ± 18$^{**\nu}$	*(28)*	130 ± 36$^{\nu}$	*(23)*	41 ± 46$^{\nu}$	*(8)*
	PoDG	58 ± 25$^{\nu}$	*(9)*	117 ± 47$^{\nu}$	*(20)*	137 ± 55$^{\nu}$	*(23)*	97 ± 47$^{\nu}$	*(15)*
**A**	CA1-SO	79 ± 15	*(9)*	141 ± 75	*(20)*	249 ± 190**	*(32)*	99 ± 64	*(12)*
	CA1-SP	31 ± 23	*(5)*	117 ± 23$^{\mu}$	*(12)*	323 ± 240$^{**\mu\rho}$	*(30)*	77 ± 50	*(10)*
	CA1-SR	64 ± 39	*(8)*	122 ± 38	*(18)*	214 ± 97*	*(31)*	79 ± 19	*(10)*
	CA2-SO	70 ± 34	*(8)*	115 ± 18$^{\nu}$	*(16)*	171 ± 63$^{\zeta\nu}$	*(27)*	96 ± 100	*(11)*
	CA2-SP	27 ± 29	*(6)*	105 ± 27$^{\nu}$	*(13)*	227 ± 78$^{**\eta}$	*(28)*	52 ± 47$^{\pi}$	*(8)*
	CA2-SR	40 ± 33	*(5)*	100 ± 20$^{\nu}$	*(14)*	92 ± 62$^{\delta\theta\nu}$	*(14)*	69 ± 21	*(9)*
	CA3-SO	33 ± 4	*(4)*	113 ± 14$^{\nu}$	*(17)*	113 ± 24$^{\varepsilon\nu}$	*(19)*	49 ± 23	*(8)*
	CA3-SP	18 ± 7	*(4)*	139 ± 53	*(18)*	110 ± 103$^{\zeta\nu}$	*(15)*	35 ± 14	*(7)*
	CA3-SR	53 ± 22	*(7)*	103 ± 30$^{\nu}$	*(15)*	127 ± 67$^{\eta\nu}$	*(19)*	63 ± 26	*(8)*
	CA3-SL	35 ± 44	*(5)*	216 ± 46$^{*\beta}$	*(27)*	110 ± 60$^{\beta\nu}$	*(16)*	77 ± 30	*(11)*
	LMOL	182 ± 75	*(16)*	329 ± 91$^{\gamma\delta\varepsilon\zeta\eta\lambda}$	*(32)*	344 ± 155$^{*\delta\zeta\eta\theta\lambda\mu\rho}$	*(34)*	162 ± 38	*(15)*
	MoDG	84 ± 33	*(10)*	164 ± 20	*(21)*	191 ± 82	*(25)*	108 ± 27	*(13)*
	GrDG	37 ± 5	*(10)*	205 ± 43*	*(28)*	188 ± 63*	*(33)*	66 ± 56.29$^{\varepsilon}$	*(15)*
	PoDG	74 ± 13	*(11)*	146 ± 61	*(25)*	149 ± 57$^{\beta\nu}$	*(26)*	97 ± 61	*(14)*
**RD**	CA1-SO	100 ± 33	*(12)*	99 ± 10	*(15)*	87 ± 5	*(12)*	134 ± 34	*(17)*
	CA1-SP	146 ± 36	*(28)*	196 ± 69$^{\gamma\nu}$	*(20)*	100 ± 41	*(11)*	139 ± 29	*(20)*
	CA1-SR	102 ± 16	*(13)*	71 ± 36$^{\beta\theta\mu}$	*(11)*	56 ± 13$^{\varepsilon\theta}$	*(8)*	116 ± 19$^{\mu}$	*(14)*
	CA2-SO	92 ± 30	*(11)*	117 ± 87	*(16)*	70 ± 54	*(9)*	119 ± 37	*(15)*
	CA2-SP	92 ± 82	*(16)*	138 ± 97	*(16)*	145 ± 47$^{\gamma\nu}$	*(18)*	120 ± 41	*(20)*
	CA2-SR	91 ± 49	*(13)*	99 ± 38	*(14)*	95 ± 64	*(15)*	120 ± 32	*(15)*
	CA3-SO	172 ± 28	*(22)*	154 ± 31	*(23)*	106 ± 50	*(16)*	125 ± 19	*(20)*
	CA3-SP	158 ± 50	*(37)*	188 ± 20$^{\gamma\nu}$	*(24)*	148 ± 45$^{\gamma\nu}$	*(22)*	163 ± 35	*(33)*
	CA3-SR	138 ± 21	*(19)*	103 ± 4	*(15)*	92 ± 34	*(14)*	139 ± 20	*(19)*
	CA3-SL	133 ± 52	*(22)*	195 ± 114$^{\gamma\nu\rho}$	*(24)*	113 ± 49	*(19)*	204 ± 14$^{\gamma\nu\xi\pi}$	*(30)*
	LMOL	74 ± 32	*(7)*	65 ± 18$^{\beta\theta\mu}$	*(6)*	54 ± 16$^{\varepsilon\theta}$	*(6)*	110 ± 36$^{\mu}$	*(10)*
	MoDG	81 ± 15	*(10)*	96 ± 18	*(12)*	69 ± 18	*(9)*	95 ± 27$^{\mu}$	*(12)*
	GrDG	163 ± 62	*(39)*	103 ± 30	*(14)*	96 ± 32	*(17)*	106 ± 20$^{\mu}$	*(30)*
	PoDG	150 ± 6	*(23)*	84 ± 41$^{\mu}$	*(16)*	81 ± 26	*(14)*	158 ± 57	*(23)*
**HR**	CA1-SO	1 ± 2	*(0)*	8 ± 9	*(1)*	11 ± 5	*(2)*	2 ± 2	*(0)*
	CA1-SP	2 ± 3	*(0)*	8 ± 8	*(1)*	10 ± 11	*(1)*	3 ± 4	*(0)*
	CA1-SR	2 ± 0	*(0)*	5 ± 2	*(1)*	15 ± 10	*(2)*	4 ± 4	*(0)*
	CA2-SO	11 ± 18	*(1)*	23 ± 24	*(3)*	9 ± 12	*(1)*	0 ± 0	*(0)*
	CA2-SP	0 ± 0	*(0)*	0 ± 0	*(0)*	6 ± 13$^{\nu}$	*(1)*	0 ± 0	*(0)*
	CA2-SR	0 ± 0	*(0)*	0 ± 0	*(0)*	0 ± 0$^{\nu}$	*(0)*	4 ± 8	*(1)*
	CA3-SO	3 ± 3	*(0)*	7 ± 7	*(1)*	3 ± 7$^{\nu}$	*(1)*	3 ± 4	*(0)*
	CA3-SP	0 ± 0	*(0)*	10 ± 4	*(1)*	11 ± 10	*(2)*	1 ± 3	*(0)*
	CA3-SR	0 ± 0	*(0)*	3 ± 4	*(0)*	5 ± 7$^{\nu}$	*(1)*	2 ± 5	*(0)*
	CA3-SL	0 ± 0	*(0)*	7 ± 12	*(1)*	10 ± 15	*(2)*	3 ± 6	*(0)*
	LMOL	6 ± 6	*(1)*	6 ± 5	*(1)*	25 ± 16$^{*\varepsilon\zeta\eta\lambda}$	*(3)*	10 ± 4	*(1)*
	MoDG	6 ± 2	*(1)*	9 ± 4	*(1)*	9 ± 5	*(1)*	6 ± 4	*(1)*
	GrDG	0 ± 0	*(0)*	17 ± 14	*(2)*	7 ± 5	*(1)*	7 ± 8	*(1)*
	PoDG	10 ± 3	*(1)*	8 ± 7	*(1)*	12 ± 21	*(2)*	7 ± 10	*(1)*

R: ramified, **H**: hypertrophic, **B**: bushy, **A**: ameoboid, **RD**: rod, **HR**: hyper-rod, **CA**: cardia arrest, **CA11**: 11 mins cardiac arrest, **CA12**: 12 mins cardiac arrest, **CA+HT**: Hypothermia treatment after 12.5 mins cardiac arrest, **CA**: cornu ammonis, **SO**: stratum oriens, **SP**: stratum pyramidale, **SR**: stratum radiatum, **SL**: stratum lucidum, **LMOL**: lacunosum moleculare of the outer layer, **MoDG**: molecular layer of the dentate gyrus, **GrDG**: granule cell layer of the dentate gyrus, **PoDG**: polymorphic layer of the dentate gyrus; *p < 0.05, **p < 0.01: vs. control; p<0.05: $^{\alpha}$vs CA1-SO, $^{\beta}$vs CA1-SP, $^{\gamma}$vs CA1-SR, $^{\delta}$vs CA2-SO, $^{\varepsilon}$vs CA2-SP, $^{\zeta}$vs CA2-SR, $^{\eta}$vs CA3-SO, $^{\theta}$vs CA3-SP, $^{\lambda}$vs CA3-SR, $^{\mu}$vs CA3-SL, $^{\nu}$vs LMOL, $^{\xi}$vs MoDG, $^{\pi}$vs GrDG, $^{\rho}$vs PoDG; all data are reported as mean ± SD.

Supplementary Table 5 | Region-specific microglial activations in thalamus following cardiac arrest and hypothermia treatment. Cell density was reported in counts per mm^2.

	Subregion	Control (n=3)		CA11 (n=3)		CA12 (n=5)		CA+HT (n=5)	
**MA Score**	VPM/L	0.12 ± 0.03		0.31 ± 0.05		0.39 ± 0.11**		0.20 ± 0.02	
	RT	0.14 ± 0.02		0.55 ± 0.12$^{**\alpha}$		0.59 ± 0.15$^{**\alpha}$		0.65 ± 0.05$^{**\alpha}$	
		count	***%***	***count***	***%***	***count***	***%***	***count***	***%***
**R**	VPM/L	360 ± 74	*(61)*	157 ± 28*	*(32)*	163 ± 45*	*(28)*	237 ± 76	*(46)*
	nRT	404 ± 84	*(58)*	210 ± 97**	*(18)*	140 ± 79**	*(14)*	136 ± 26**	*(12)*
**H**	VPM/L	26 ± 17	*(7)*	24 ± 7	*(5)*	37 ± 18	*(6)*	23 ± 14	*(4)*
	nRT	35 ± 24	*(5)*	67 ± 32$^{\alpha}$	*(6)*	48 ± 12	*(5)*	55 ± 10$^{\alpha}$	*(5)*
**B**	VPM/L	36 ± 22	*(6)*	74 ± 16	*(15)*	100 ± 32	*(17)*	53 ± 13*	*(11)*
	nRT	48 ± 18	*(7)*	337 ± 57$^{**\alpha}$	*(30)*	244 ± 76$^{**\alpha}$	*(23)*	338 ± 72$^{**\alpha}$	*(29)*
**A**	VPM/L	38 ± 6	*(7)*	92 ± 27	*(18)*	136 ± 44	*(24)*	55 ± 8	*(11)*
	nRT	49 ± 8	*(7)*	354 ± 113$^{**\alpha}$	*(31)*	417 ± 154$^{**\alpha}$	*(40)*	494 ± 59^{**a}	*(43)*
**RD**	VPM/L	129 ± 20	*(22)*	138 ± 29	*(28)*	126 ± 31	*(22)*	135 ± 13	*(27)*
	nRT	154 ± 17	*(23)*	139 ± 66	*(12)*	155 ± 82	*(15)*	110 ± 56	*(10)*
**HR**	VPM/L	1 ± 1	*(0)*	8 ± 3	*(2)*	13 ± 7	*(2)*	4 ± 1	*(1)*
	RT	4 ± 2	*(1)*	31 ± 12$^{**\alpha}$	*(3)*	30 ± 9$^{**\alpha}$	*(3)*	26 ± 11$^{**\alpha}$	*(2)*

MA: microglial activation, **R**: ramified, **H**: hypertrophic, **B**: bushy, **A**: ameoboid, **RD**: rod, **HR**: hyper-rod, **CA**: cardia arrest, **CA11**: 11 mins cardiac arrest, **CA12**: 12 mins cardiac arrest, **CA+HT**: Hypothermia treatment after 12.5 mins cardiac arrest; *$p < 0.05$, **$p < 0.01$: vs. control; $^{\alpha}p < 0.05$: VPM/L vs nRT; all data are reported as mean ± SD.

Supplementary Table 6 | Region-specific microglial activations in the brain following cardiac arrest and hypothermia treatment. Cell density was reported in counts per mm^2.

	**Division**	**Control (n=3)**		**CA11 (n=3)**		**CA12 (n=5)**		**CA+HT (n=5)**	
**MA Score**	Whole Brain	0.22 ± 0.06		0.34 ± 0.02		0.44 ± 0.09**		0.26 ± 0.03$^{\delta}$	
	Cerebrum	0.23 ± 0.07		0.36 ± 0.01		0.45 ± 0.09**		0.24 ± 0.03$^{\delta}$	
	Brain Stem	0.13 ± 0.03		0.23 ± 0.06		0.37 ± 0.13**		0.22 ± 0.03$^{\delta}$	
	Cerebellum	0.19 ± 0.05		0.36 ± 0.03		0.45 ± 0.16**		0.47 ± 0.14$^{**\alpha\beta\gamma}$	
		count	***%***	***count***	***%***	***count***	***%***	***count***	***%***
**R**	Whole Brain	6,171 ± 1269$^{\delta}$	*(51)*	4,317 ± 654	*(36)*	3,692 ± 1,225$^{*\delta}$	*(28)*	6,093 ± 527$^{\delta}$	*(46)*
	Cerebrum	6,707 ± 1704$^{\delta}$	*(51)*	4,699 ± 732$^{\delta}$	*(36)*	4,032 ± 1,267$^{**\delta}$	*(28)*	7,070 ± 690$^{\delta}$	*(48)*
	Brain Stem	7,218 ± 223$^{\delta}$	*(62)*	4,878 ± 1570$^{*\delta}$	*(47)*	4,617 ± 1871$^{**\delta}$	*(34)*	6,366 ± 457$^{\delta}$	*(50)*
	Cerebellum	3,165 ± 327$^{\alpha\beta\gamma}$	*(43)*	2,293 ± 426$^{\beta\gamma}$	*(28)*	1,618 ± 958$^{\alpha\beta\gamma}$	*(20)*	1,665 ± 596$^{\alpha\beta\gamma}$	*(23)*
**H**	Whole Brain	1,158 ± 196$^{\gamma\delta}$	*(10)*	1,019 ± 252$^{\delta}$	*(9)*	1,276 ± 464$^{\delta}$	*(9)*	1,312 ± 274$^{\delta}$	*(10)*
	Cerebrum	1,491 ± 239$^{\gamma\delta}$	*(11)*	1,269 ± 314$^{\gamma\delta}$	*(10)*	1,536 ± 590$^{\gamma\delta}$	*(10)*	1,606 ± 353$^{\gamma\delta}$	*(11)*
	Brain Stem	440 ± 143$^{\alpha\beta}$	*(4)*	459 ± 147$^{\beta}$	*(4)*	976 ± 297$^{\beta\delta}$	*(7)*	841 ± 202$^{\beta\delta}$	*(7)*
	Cerebellum	166 ± 45$^{\alpha\beta}$	*(2)*	289 ± 57$^{\alpha\beta}$	*(4)*	335 ± 58$^{\alpha\beta\gamma}$	*(4)*	335 ± 118$^{\alpha\beta\gamma}$	*(4)*
**B**	Whole Brain	1,585 ± 524	*(13)*	2,324 ± 210	*(20)*	3,380 ± 1257$^{**\delta}$	*(25)*	1,986 ± 422	*(15)*
	Cerebrum	1,974 ± 655	*(15)*	2,811 ± 240$^{\gamma\delta}$	*(22)*	4,036 ± 1628$^{**\gamma\delta}$	*(27)*	2,242 ± 467	*(15)*
	Brain Stem	660 ± 220	*(6)*	983 ± 260$^{\beta}$	*(10)*	2,254 ± 605$^{*\beta}$	*(17)*	1,371 ± 265	*(11)*
	Cerebellum	488 ± 153	*(7)*	1,068 ± 88$^{\beta}$	*(13)*	1,248 ± 238$^{\alpha\beta}$	*(16)*	1,264 ± 530	*(17)*
**A**	Whole Brain	1,096 ± 228	*(9)*	2,108 ± 498	*(18)*	3,170 ± 1181**	*(24)*	1,604 ± 308	*(12)*
	Cerebrum	1,165 ± 231	*(9)*	2,228 ± 633	*(17)*	3,334 ± 1304**	*(23)*	1,444 ± 213	*(10)*
	Brain Stem	891 ± 186	*(8)*	1,487 ± 418	*(15)*	2,919 ± 1185**	*(22)*	1,504 ± 256	*(12)*
	Cerebellum	929 ± 246	*(13)*	1,984 ± 33	*(25)*	2,616 ± 838*	*(35)*	2,368 ± 922	*(32)*
**RD**	Whole Brain	1,939 ± 212	*(16)*	1,970 ± 314	*(17)*	1,693 ± 355	*(13)*	2,077 ± 124	*(16)*
	Cerebrum	1,731 ± 275	*(13)*	1,829 ± 505	*(14)*	1,609 ± 409$^{\gamma}$	*(11)*	2,125 ± 183	*(15)*
	Brain Stem	2,427 ± 80	*(21)*	2,385 ± 395	*(23)*	2,299 ± 478$^{\beta\delta}$	*(17)*	2,525 ± 177	*(20)*
	Cerebellum	2,531 ± 224	*(34)*	2,317 ± 451	*(28)*	1,670 ± 759$^{*\gamma}$	*(21)*	1,578 ± 514**	*(21)*
**HR**	Whole Brain	74 ± 24	*(1)*	148 ± 17	*(1)*	218 ± 67**	*(2)*	152 ± 34$^{\delta}$	*(1)*
	Cerebrum	77 ± 22	*(1)*	140 ± 28	*(1)*	205 ± 70*	*(1)*	129 ± 27$^{\delta}$	*(1)*
	Brain Stem	45 ± 15	*(0)*	86 ± 13$^{\delta}$	*(1)*	210 ± 87**	*(2)*	113 ± 25$^{\delta}$	*(1)*
	Cerebellum	82 ± 36	*(1)*	218 ± 27$^{*\gamma}$	*(3)*	280 ± 68**	*(4)*	278 ± 92$^{**\alpha\beta\gamma}$	*(4)*

MA: microglial activation, **R**: ramified, **H**: hypertrophic, **B**: bushy, **A**: ameoboid, **RD**: rod, **HR**: hyper-rod, **CA**: cardia arrest, **CA11**: 11 mins cardiac arrest, **CA12**: 12 mins cardiac arrest, **CA+HT**: Hypothermia treatment after 12.5 mins cardiac arrest; *$p < 0.05$, **$p < 0.01$: vs. control; $p < 0.05$: $^{\alpha}$vs Whole Brain, $^{\beta}$vs Cerebrum, $^{\gamma}$vs Brain Stem, $^{\delta}$vs Cerebellum; all data are reported as mean ± SD.
