# Supplementary material for: StainAI: quantitative mapping of stained microglia and insights into brain-wide neuroinflammation and therapeutic effects in cardiac arrest
Source: Commun Biol. 2025 Mar 20;8:462. doi: 10.1038/s42003-025-07926-y (PMC11926354; doi:10.1038/s42003-025-07926-y)
Supplement: Supplementary file 2 — Description of Additional Supplementary Materials [file 42003_2025_7926_MOESM2_ESM.docx]

**Description of Additional Supplementary Files**

**File name:** Supplementary Data 1

**Description:**

The provided Excel sheets contain raw data details and additional statistical analyses corresponding to the figures in the study.

*Table 3:* StainAI-Groundtruth classification confusion matrix and statistical analysis.

*Figure 4A, Supplementary Table 2:* Data values for total cell density, microglial activation (MA) score, and densities of ramified, hypertrophic, bushy, amoeboid, rod, and hyper-rod cells in the primary somatosensory cortical layers (Layer 1, 2/3, 4, 5, and 6).

*Figure 4B, Supplementary Table 3, 4:* Data values for total cell density, MA score, and densities of ramified, hypertrophic, bushy, amoeboid, rod, and hyper-rod cells in hippocampal sub-regions, including:

- CA1, CA2, CA3: Stratum oriens (SO), stratum pyramidale (SP), stratum radiatum (SR), and stratum lucidum (SL).
- Dentate gyrus: Lacunosum moleculare of the outer layer (LMOL), molecular layer (MoDG), granule cell layer (GrDG), and polymorphic layer (PoDG).

*Figure 4C, Supplementary Table 5:* Data values for total cell density, MA score, and densities of ramified, hypertrophic, bushy, amoeboid, rod, and hyper-rod cells in the somatosensory thalamus, including the thalamic reticular nucleus (nRT) and ventral posterior medial/lateral nucleus (VPM/L).

*Figure 5:* Data values for total cell density, MA score, and densities of ramified, hypertrophic, bushy, amoeboid, rod, and hyper-rod cells across the whole brain, cerebrum, cerebellum, and brainstem.
